# From Natural Products to New Synthetic Small Molecules: A Journey through the World of Xanthones

**DOI:** 10.3390/molecules26020431

**Published:** 2021-01-15

**Authors:** Madalena M. M. Pinto, Andreia Palmeira, Carla Fernandes, Diana I. S. P. Resende, Emília Sousa, Honorina Cidade, Maria Elizabeth Tiritan, Marta Correia-da-Silva, Sara Cravo

**Affiliations:** 1 Laboratory of Organic and Pharmaceutical Chemistry (LQOF), Department of Chemical Sciences, Faculty of Pharmacy, University of Porto, Rua de Jorge Viterbo Ferreira, 228, 4050-313 Porto, Portugal; andreiapalmeira@gmail.com (A.P.); cfernandes@ff.up.pt (C.F.); dresende@ff.up.pt (D.I.S.P.R.); esousa@ff.up.pt (E.S.); hcidade@ff.up.pt (H.C.); beth@ff.up.pt (M.E.T.); m_correiadasilva@ff.up.pt (M.C.-d.-S.); scravo@ff.up.pt (S.C.); 2 Interdisciplinary Centre of Marine and Environmental Research (CIIMAR), Terminal de Cruzeiros do Porto de Leixões, Av. General Norton de Matos s/n, 4450-208 Matosinhos, Portugal; 3 CESPU, Instituto de Investigação e Formação Avançada em Ciências e Tecnologias da Saúde, Rua Central de Gandra, 1317, 4585-116 Gandra PRD, Portugal

**Keywords:** xanthones, thioxanthones, marine natural products, biological activities, synthesis, chiral

## Abstract

This work reviews the contributions of the corresponding author (M.M.M.P.) and her research group to Medicinal Chemistry concerning the isolation from plant and marine sources of xanthone derivatives as well as their synthesis, biological/pharmacological activities, formulation and analytical applications. Although her group activity has been spread over several chemical families with relevance in Medicinal Chemistry, the main focus of the investigation and research has been in the xanthone family. Xanthone derivatives have a variety of activities with great potential for therapeutic applications due to their versatile framework. The group has contributed with several libraries of xanthones derivatives, with a variety of activities such as antitumor, anticoagulant, antiplatelet, anti-inflammatory, antimalarial, antimicrobial, hepatoprotective, antioxidant, and multidrug resistance reversal effects. Besides therapeutic applications, our group has also developed xanthone derivatives with analytical applications as chiral selectors for liquid chromatography and for maritime application as antifouling agents for marine paints. Chemically, it has been challenging to afford green chemistry methods and achieve enantiomeric purity of chiral derivatives. In this review, the structures of the most significant compounds will be presented.

## 1. Introduction

### 1.1. Why Choose “Xanthone Derivatives”?

#### 1.1.1. Xanthone: The Molecule

**Figure 1:**
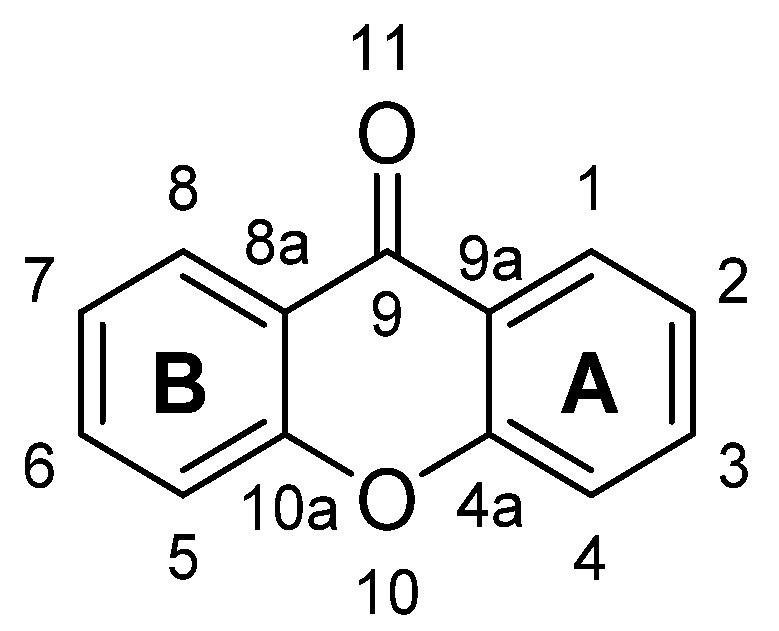
Structure of xanthone (9*H*-xanthen-9-one).

Xanthone is an aromatic oxygenated heterocyclic molecule, with a dibenzo-γ-pirone scaffold, known as 9*H*-xanthen-9-one, with the molecular formula of C_13_H_8_O_2_ (Figure 1). The numbering and designation of rings A and B come from the biosynthetic pathways for the compounds from higher plants, A-ring (carbons 1–4) being acetate-derived whereas the shikimic acid pathway gives B-ring (carbons 5–8); the other carbon atoms are numbered according IUPAC 2004 recommendations for structure elucidation purposes.

**Scheme 1:**
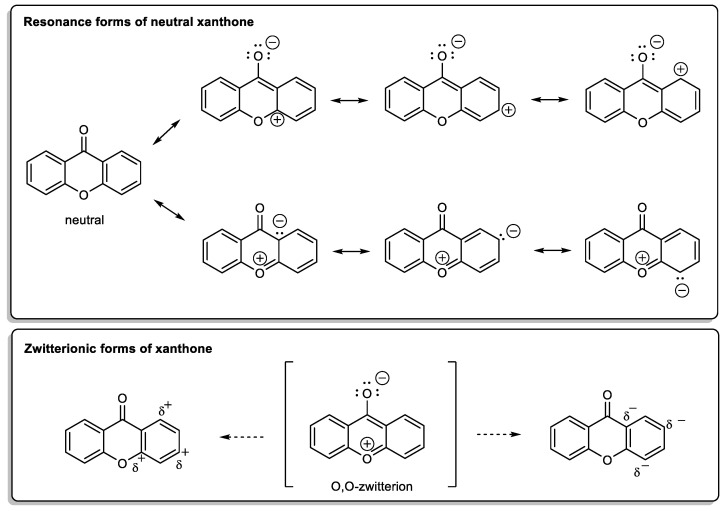
Resonance forms of xanthone molecule.

X-Ray diffraction data are an important tool, not only for structure elucidation but also to help in understanding the mechanism of action of the wide range of biological and pharmacological activities showed by xanthone derivatives. The crystal structure of 9*H*-xanthen-9-one (Figure 1) was first reported in 1982 [1] and later, using more accurate experimental techniques, the data have been improved [2,3]. Considering the molecule of xanthone itself in solid state, it is essentially planar due to the conjugated ring systems, except for the O(11) atom, which deviates 0.13 Å from the plane, with the central pyranoid ring with partial aromatic character. Due to the tricyclic-fused ring system, free rotation is limited. The rigidity of this scaffold contributes to the stability of the compound. For xanthone derivatives, slight differences can be found dependent on the nature of the substituents and their localization on the scaffold. The three-ring system can be slightly twisted along its longitudinal axis due to steric factors associated with the substituents, especially for bulky groups [3]. Chemically, the xanthone core looks very simple but can display a very rich reactivity profile, which results mainly from the resonance forms (Scheme 1). The oxygen atoms belonging to the biaryl ether group and the carbonyl group are involved in those forms; the planar tricyclic system, associated with a zwitterionic form, contributes for the poor solubility of the xanthone itself, which can be changed by adding substituents, concurring to higher solubility.

#### 1.1.2. A New Molecule Was Born from the Lab and from the Nature

**Scheme 2:**
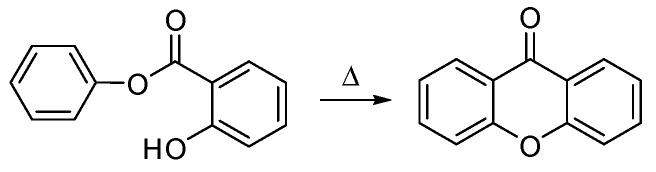
Xanthone synthesis from phenyl salicylate proposed by Holleman and verifed by Clarke [4,9].

9*H*-Xanthen-9-one (Figure 1) is not a natural product, in fact, this structure was only obtained by synthesis in 1860, by Kolbe and Lautermann, using phosphorus oxychloride in sodium salicylate via condensation of phenol and salicylic acid [4]. Historically, the first natural xanthone described was gentisin (1,7-dihydroxy-3-methoxyxanthone) in 1821, isolated from the plant *Gentiana lutea* [5] and the first prenylxanthone derivative, tajixanthone, was isolated from the mycelium of *Aspergillus stellatus* in 1970 [6]. However, the first synthesis proposed for a xanthone (hydroxyxanthone) was achieved by Michael, in 1883, and later by Kostanecki and Nessler, in 1891, through the distillation of *O*-hydroxy-benzoic acid, acetic anhydride and a phenol [7,8], or by heating phenyl salicylate alone [4,9] (Scheme 2), while the first total synthesis of a naturally occurring xanthone was described by Ullmann and Panchaud, in 1906, for euxanthone [10].

**Figure 2:**
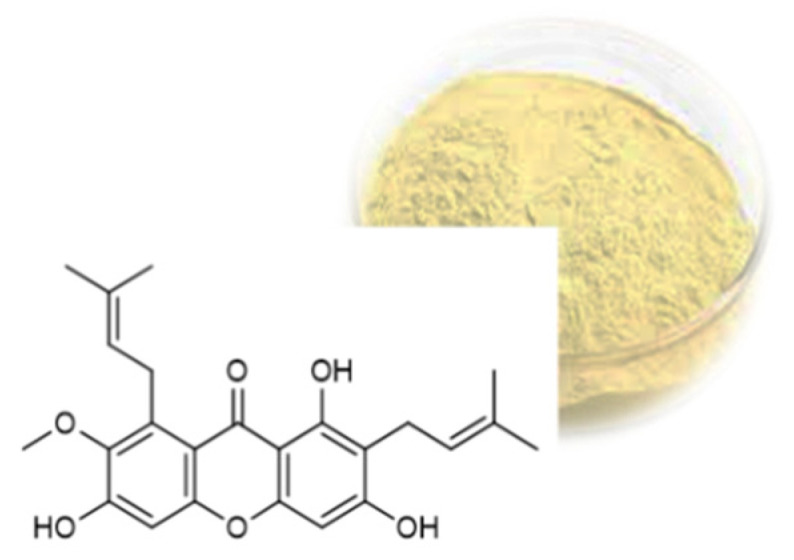
Structure of α-mangostin and the yellow colour of the compound isolated from the *Garcinia mangostana* Linn.

The designation “xanthone” was coined by Schmid due to the yellow colour of the compound isolated from the pericarp of Mangosteen (*Garcinia mangostana* Linn.), a tropical fruit belonging to the Guttiferae family, which derives from the Greek word “xanthos” (yellow) (Figure 2) [11,12].

Due to the great diversity of substituents, as well as the discovery and synthesis of new xanthones, their classification by groups has evolved. Currently, they can be divided into six major groups: simple xanthones, xanthone glycosides, prenylated xanthones, xanthonolignoids, bisxanthones, and miscellaneous xanthones [13].

A large amount of research has been carried out, not only regarding isolation from terrestrial and marine sources, but also concerning compounds obtained by synthesis and with a large diversity of applications in medicinal, analytical and environmental chemistry. In 2005, one of us was Guest Editor of a Special issue of Current Medicinal Chemistry [14], which covered papers from isolation, synthesis, magnetic resonance spectroscopy and crystal X-ray studies of several xanthone derivatives to papers covering their biological activities and mechanisms of action [3,15,16,17,18,19].

#### 1.1.3. Xanthone: A Privileged Scaffold

According the concept of Evans et al. [20] the xanthone nucleus could be considered as a “privileged structure”, taking into account its binding to multiple, unrelated classes of protein receptors as high affinity ligands. Following, several authors associated the “xanthone” to the “privileged structure concept” [21,22,23,24,25]. This ability of xanthones to interfere with a variety of biological targets is related with some special molecular features such as the presence of a heteroaromatic tricyclic ring system predominantly planar and rigid, a carbonyl group at the central ring able of several interactions, a biaryl ether group contributing to the electronic system, and the xanthone core that accommodates a vast variety of substituents at different positions.

## 2. A Library of Natural Mimetic Xanthones Looking for Biological Diversity: From the Land and from the Sea

Our journey through the world of xanthones comprises several stages and objectives. To obtain a library of natural mimetic xanthones we have been working in an interactive way including isolation of new compounds, total synthesis or molecular modifications, screening of biological activities and in-deep studies regarding their absorption, distribution, metabolism, excretion, toxicity (ADMET) properties as well as formulation of new compounds with appropriated drug delivery systems.

Natural xanthones are secondary metabolites that may be found in higher plants, lichens and fungi from terrestrial and marine origins [13,26,27,28,29,30,31]. As a whole, we can say that the “meeting point” of our work is bioinspiration. The synthesis of non-natural nature-mimetic small molecules could be an opportunity of giving Nature a helping-hand in generating new bioactive agents and enlarging chemical-biological space. Following the idea of mimic Nature, a common approach of most medicinal chemistry programs it is crucial: (i) the choice of scaffolds, and (ii) the choice of strategies for molecular modifications. From our point of view, bioinspiration is very useful for selection of molecules and scaffolds as well as for selection of synthetic strategies.

Many products from marine origin have been described, and every year hundreds of new compounds are being discovered. The main sources of marine secondary metabolites with interest in medicinal chemistry are marine invertebrates such as sponges, tunicates, mollusks, and bryozoans, as well as algae and marine microorganisms, such as cyanobacteria and fungi. The designated “marine-derived fungi” must grow and sporulate in a marine environment, while facultative marine fungi are from terrestrial or freshwater habitats, but may also grow in the marine environment [32]. Considering that marine organisms are a very important source of new bioactive molecules with a plethora of biological activities with the antimicrobial being one of the most relevant for medicinal chemistry.

Xanthones are not frequently mentioned as marine natural products due to the relatively late interest on marine microorganisms as providers of bioactive secondary metabolites comparing with terrestrial sources. For this reason, marine-derived fungi have emerged as a potential source of marine natural products and similar to their terrestrial counterparts, marine-derived fungi also produce xanthones. Many xanthones sharing the same structure can be found, for example, from both marine and terrestrial fungi while structures that are more complex have been found only in marine-derived fungi and actinomycetes. Comparing with other chemical families we can say that the number of xanthones isolated from marine sources is quite scarce. Xanthones were found predominantly in marine-derived fungi from the genera *Aspergillus*, *Penicillium*, *Monodictys*, *Emericella*, *Phoma*, *Paecilomyces*, *Chaetomium*, *Wardomyces* and from marine-derived *Actinomycetes* (*Actinomadura* and *Streptomyces*) [30]. From these data we can infer that the “marine xanthones” obey the same rule as their terrestrial analogues concerning the large variety of biological activities, with greater emphasis on antimicrobial and antitumor activities. More recently, a review from our group (between 1989 and 2018) [27], emphasizes the anti-infective activity (antibacterial, antifungal, antiparasitic, and antiviral) of 53 xanthones from the marine environment; furthermore, molecular descriptors, biophysicochemical properties, and pharmacokinetic parameters were calculated for three sets of compounds (xanthones, hydroxyxanthones, and glycosylated derivatives) and the chemical space occupied by marine xanthone derivatives was compared with marketed drugs. From our data it can be inferred that xanthone derivatives have a good compliance with the drug-likeness chemical space. Hydroxyxanthones and glycosylated derivatives exceed the desired values and a poor pharmacokinetic behavior may be predicted [27].

**Figure 3:**
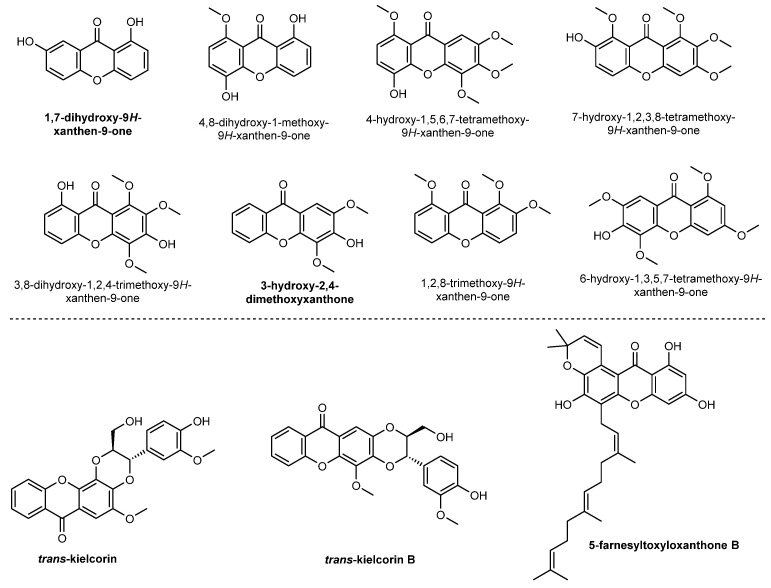
Terrestrial natural xanthones isolated by our research group.

Xanthones with very different structures can be found in higher plants, lichens and bacteria, that have been reviewed since 1961 [33] until recently [13,15,23,34]. Figure 3 shows some representative xanthones isolated by our group [35,36,37,38,39,40].

In this work, we describe xanthone derivatives divided in seven groups, based on different chemical families, concerning the diverse substituents. We will exemplify strategies that we consider significant for our contribution: prenylation, sulfation, and introduction of chiral moieties starting from simple oxygenated xanthones. Given the objective of this work, most of the references indicated throughout the text correspond mainly to the contribution of our group to medicinal chemistry in the chemical family of xanthones and not to an exhaustive research in this area. We expect that this review may serve as an inspiration for other groups working with diverse “privileged structures” in an integrative way.

### 2.1. Simple Oxygenated Xanthones

Simple oxygenated xanthones are xanthones with simple substituents such as hydroxyl, methoxy or methyl. This group is abundant in many natural products [15] and are starting points for many synthetic xanthones [16]. As simple phenolic compounds, naturally-occurring simple oxygenated xanthones have been described for their antioxidant properties that have been implicated in their hepatoprotective, anti-inflammatory, and cancer chemopreventive actions [18]. The influence of these substituents in the xanthone stability is important to understand the biological effects of these compounds [41]. Our group has been gathering physical-chemical-biological properties of these simple derivatives to use this information in drug design of more complex xanthones.

Early in 1997, a small library of 12 mono and trioxygenated xanthones was investigated for their influence on the complement system and revealed anticomplementary activity of 3-hydroxy- and 4-hydroxyxanthones [42], probably based on chelation of Ca^2+^. This library of simple oxygenated xanthones enlarged over the years both from natural [36,38,39] and synthetic derivatives, to more than 60 simple oxygenated xanthones and hundreds of more complex derivatives with substituents at different positions. This collection of xanthones allowed to establish structure-NMR chemical shift relationships [43] and gather X-ray crystallographic data [44,45] to guide synthetic chemists. Also, this appendance diversity-oriented synthesis allowed to develop a chemical toolbox of synthetic conditions and intermediates, based on classical and novel methods to obtain xanthones, recently reviewed in [46] and to establish methods for their quantification validated in a diversity of matrices [47].

**Figure 4:**
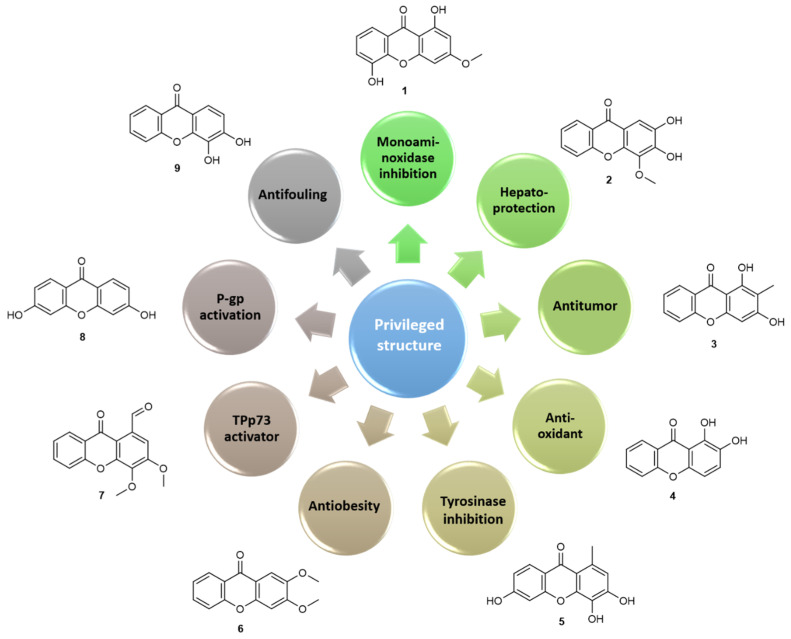
Hit compounds from simple oxygenated xanthones for several activities and molecular targets.

This chemical collection of pure xanthones (>95%) was submitted to several screenings to discover their biological activities which allowed to build quantitative structure–activity relationship [(Q)SAR] models and to establish pharmacophores for antitumor [48], antioxidant [49], hepatoprotection [50], antifungal [29,51], antibacterial [52,53], antiobesity [54], antifouling [55] activities and/or targets such as monoaminoxidase (MAO) [56], P-glycoprotein (P-gp) [57,58], protein kinase C (PKC) [59,60], tyrosinase [61], over these two last decades. Moreover, from these studies several simple oxygenated (Figure 4) or more complex hit compounds emerged that were additionally investigated in many directions, namely for further molecular modifications [62], for their cellular and molecular mechanism of actions [63,64], for their ADMET properties [65,66,67], for chromatographic applications [68,69] and for their incorporation in nanoformulations [70,71].

The ethnopharmacological relevance of *Hypericum perforatum* extracts as antidepressant envisioned a first screening of simple oxygenated xanthones on targets related to central nervous system (CNS) disorders. On MAO studies, among 53 simple natural and synthetic xanthones, most of the compounds acted preferentially as MAO-A competitive, reversible inhibitors with IC_50_ values in the micro- to nanomolar range. Compound 1,5-dihydroxy-3-methoxyxanthone (**1**) with an IC_50_ of 40 nM for MAO-A emerged as the most active inhibitor. A surprising result was the strong activity and selectivity noted for the unsubstituted xanthone while hexasubstitution was an unfavourable feature for this effect. With this data, it was possible to build a 3D-QSAR model, which revealed the importance of the distance between two H-bond-acceptor groups in modulating this activity. Similar working hypothesis was made to investigate the hepatoprotective of xanthonolignoids (detailed in a following section), inspired in the hepatoprotective lignoid sylibin [50]. Along with xanthonolignoids, their synthetic intermediates were also investigated and 3,4-dihydroxy-2-methoxyxanthone and 2,3-dihydroxy-4-methoxyxanthone (**2**) were found to be even more potent than the investigated lignoids.

The tricyclic scaffold and the chemopreventive activity of naturally-occurring xanthones were also the reason to investigate this collection of simple oxygenated xanthones on antitumor screening activity. The in vitro effect of 27 xanthone derivatives on the growth of human cancer cell lines, led to select the naturally-occurring 1,3-dihydroxy-2-methylxanthone (**3**) that was further investigated in nanoparticle formulations to improve its activity [70]. One other hit that emerged from this study was 1,2-dihydroxyxanthone (**4**), which was initially considered promising for its effect against melanoma [48]. Due to its catechol structure *peri* to carbonyl, this compound was the most promising antioxidant agent within this collection, that was further characterized for its chelating properties, its stability, phototoxicity, cytotoxic effect on a human keratinocyte cell line [49] and its modulatory effects on the activity of the THP-1 macrophage cell line, namely cytokine production [64]. The efficacy and safety profile of xanthone **4** with potential for skin care application deserves to be further explored. The investigation of tyrosinase as a molecular target, led to discover very recently, 1-methyl-3,4,6-trihydroxyxanthone (**5**) as a potent tyrosinase uncompetitive inhibitor [61] and to stablish a QSAR model in which the partial negative surface area, the relative number of oxygen atoms, and the substitution pattern of the xanthonic core, were found to contribute to the tyrosinase inhibitory activity.

Similarly, for antiobesity activity, the number of rings, relative number of oxygen atoms, average structural information content, and partial negative surface area, among others, were positive descriptors for an increase of lipid reducing activity of xanthone derivatives [54]. In a screening of 85 polyphenols, 2,3-dimethoxyxanthone (**6**) was the hit compound showing strong lipid reducing activity in zebrafish larvae in vivo and with no general toxicity being found to reduce the mRNA expression of fatty acid synthase.

Insights into the molecular targets for antitumor activity revealed simple oxygenated xanthone as promising PKC modulators, using an in vivo yeast phenotypic assay. Methoxylated xanthone derivatives were found to be promising PKC activators showing high selectivity for individual PKC isoforms, proving their utility for a detailed study of the physiological and pathophysiological roles of PKC isoforms [59]. In contrast, simple oxygenated xanthones, with an aldehyde in position C-1, were found to be potent inhibitors of PKC [60,63]. This result led us to explore this type of substituent in simple oxygenated xanthones and to discover the inhibitor of the TAp73 interaction with MDM2 and mutant p53 with promising antitumor activity against neuroblastoma, 1-carbaldehyde-3,4-dimethoxyxanthone (**7**). This xanthone was able to release TAp73 from its interaction with both MDM2 and mutant p53, enhancing TAp73 transcriptional activity, cell cycle arrest, and apoptosis in p53-null and mutant p53-expressing tumor cells [63]. Putative metabolites with 1-(hydroxymethyl)- or carboxylic acid at position C-1 were inactive and since this compound was found to be a TAp73 activator, we investigated its antitumor potential towards neuroblastoma, discovering its potent effect in patient-derived neuroblastoma cells, both alone and in combination with conventional chemotherapeutics [63]. Very recently, xanthone **7** was encapsulated in nanostructured lipid carriers (NLCs) by ultrasonication, obtaining final loaded NLCs with mean particle sizes suitable for topical application and high encapsulation efficiencies. These loaded formulations seemed to be more cytotoxic against melanoma A375 cell line than unloaded NLCs, which indicates the potential use of NLCs as a carrier for this Tap73 activator with potent antiproliferative effect on melanoma A375 cell lines, improving its efficacy [72].

As mentioned, while performing biological activity studies, several drug-like properties (physico-chemical parameters, stability, photostability, intestinal permeability, albumin binding, toxicity, etc.) were simultaneously investigated [66,73]. While showing favourable permeability parameters, the ability of dihydroxylated xanthones for P-gp activation was disclosed with 3,6-dihydroxyxanthone (**8**) being the most active. Further studies pointed their putative application as antidotes of paraquat, by protection against the cytotoxicity induced by this pesticide P-gp substrate [57,58,74].

Beyond pharmacological applications, the antifouling (AF) activity of this collection of simple oxygenated xanthones was tested following our previous results concerning the AF activity of a sulfated and glycosylated xanthone (Section 2.4). This screening highlighted 3,4-dihydroxyxanthone (**9**) among this collection with in vivo activity toward the settlement of *Mytilus galloprovincialis* larvae (EC_50_ = 11.53 µM) and low toxicity to this macrofouling species (LC_50_ > 500 µM and LC_50_/EC_50_ = 43) and to a non-fouling species, the brine shrimp *Artemia salina* (<10% mortality at 50 µM). Xanthone **9** induced alterations in the mussel plantigrade larvae proteome, particularly toward the functions of cytoskeleton, chaperone-mediated regulation of protein activity, and cell redox status [55].

Finally, as antimicrobial agents or adjuvants, simple oxygenated xanthones are inactive or less active than other derivatives with halogens and amine substituents [29,51,53]. These studies will be detailed in following sections.

Overall, most of the discover hits were trioxygenated derivatives (Figure 4), manly with antitumor activity. Dihydroxyxanthones, particularly those with a catechol moiety, were found to be the most promising hits for activities in which redox mechanisms are involved. From a medicinal chemistry perspective, these catechol hits could be considered as PAINS, pan-assay interference compounds [75]. Although their activity does not depend on a specific, drug-like interaction between the molecule and a protein, they are able to coat a protein or sequester metal ions that are essential to a protein’s function, and these are mechanisms recognized for some FDA approved-drugs. Moreover, a screening hit compound is useful when supported by strong SAR complemented by hit-to-lead optimization [76], that we will further explore in the next sections. In fact, these simple xanthones derivatives were starting points to increase complexity and lipophilicity allowing hit optimization.

### 2.2. Prenylated Xanthones

**Figure 5:**
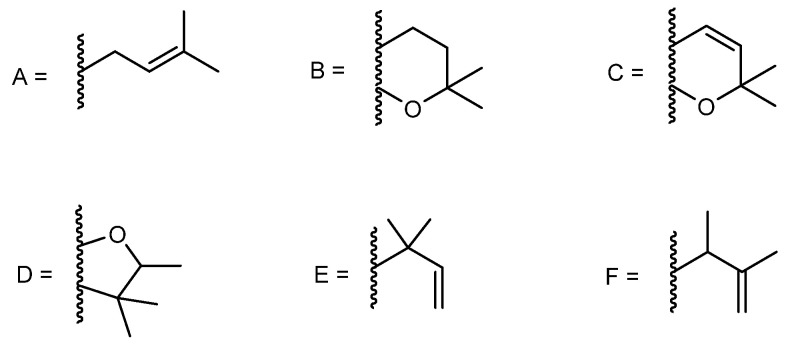
Prenyl groups found in prenylated xanthones reported by our group.

Prenylated xanthones are the major group of naturally occurring xanthones. The majority of reported synthetic prenylated xanthones possess a 3-methylbut-2-enyl or isoprenyl group (A, Figure 5). Nevertheless, the presence of other open chain prenyl groups can also be found in literature; compounds containing 2,2-dimethyldihydropyran (B), 2,2-dimethylpyran (C) and 2,3,3-trimethyldihydrofuran (D) groups, which are the result of cyclization of the prenyl substituents with the *ortho* hydroxyl group could also be found (Figure 5) [77]. Among these substitution patterns, our group has been involved in the synthesis of bioactive prenylxanthones with isoprenyl (A), 2,2-dimethyldihydropyran (B), 2,2-dimethylpyran (C), 2,3,3-trimethyldihydrofuran (D), 1,1-dimethylallyl (E), and 3-methylbut-3-en-2-yl (F) and groups (Figure 5).

**Scheme 3:**
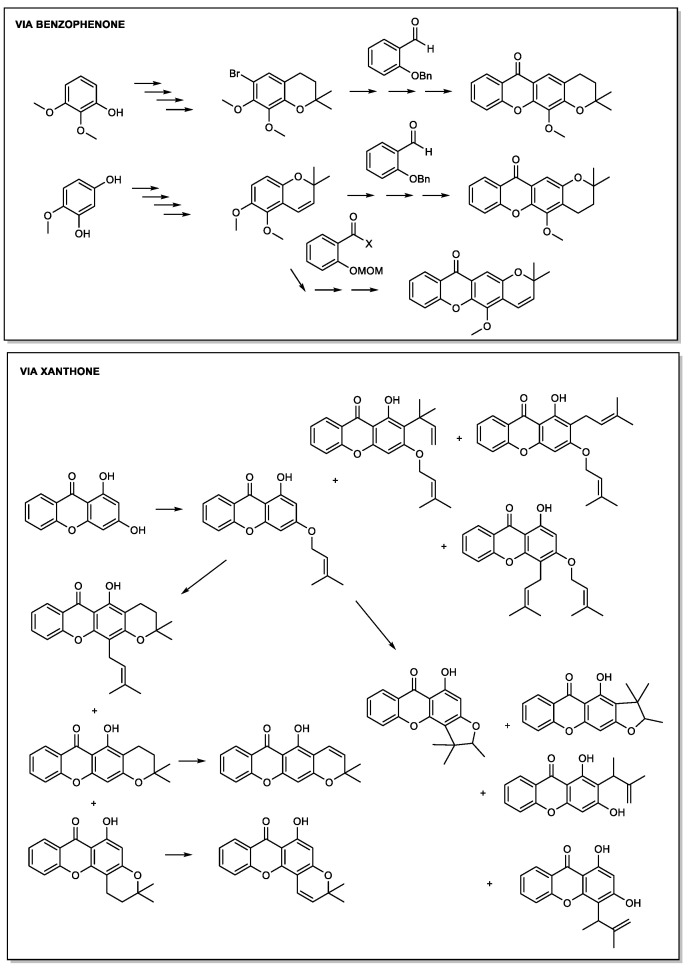
Examples of the main synthetic approachs to obtain prenylated xanthones using classical and “non-classical” methodologies, reported by our group.

Xanthones with open chain prenyl groups, as well as dihydropyranoxanthones and pyranoxanthones, were firstly obtained by our group through classical synthetic methodologies. Prenylation was carried out from hydroxyxanthones by a nucleophilic substitution reaction. Dihydropyranoxanthones were synthesized by cyclization of mono-oxyprenylated xanthones, while the synthesis of pyranoxanthones was achieved by dehydrogenation of dihydropyranoxanthones or using a benzopyran derivative as building block (Scheme 3).

Following, our group used “non-classical” methodologies, namely microwave-assisted organic synthesis (MAOS) and heterogeneous catalysis, to optimize the synthetic approach to obtain this group of xanthones. It was also applied the combined method of heterogeneous catalysis with MW irradiation [78,79,80,81]. MW methodology was applied for the first time to the synthesis of these derivatives, being obtained several prenylated xanthones with better yields, lower reaction times and higher selectivity when compared to conventional synthesis [78,79,80,81]. Heterogeneous catalysis methodology was also used for the first time, in the synthesis of dihydropyranoxanthones. By this method, dihydropyranoxanthones were obtained from hydroxyxanthones, through a one-pot synthesis, in the presence of montmorillonite K10 clay as solid catalyst. The coupling of heterogeneous catalysis with MW irradiation provided a chemical process with several advantages when compared to classical methods, such as enhanced reaction rates, better yields and selectivity to obtain xanthones with an extra dihydropyran ring [79].

The presence of prenyl groups in the xanthone nucleus can influence the physicochemical properties, namely lipophilicity. Additionally, these groups also have impact in the biological activity, as they affect the three-dimensional properties of the xanthone and consequently the interaction with the biological targets [82]. In fact, a relationship between activity and the presence of prenyl groups in key-positions on the xanthone nucleus was associated with some biological activities, such as antitumor, anti-inflammatory and human lymphocyte proliferation inhibitory effects [34,38,83].

**Figure 6:**
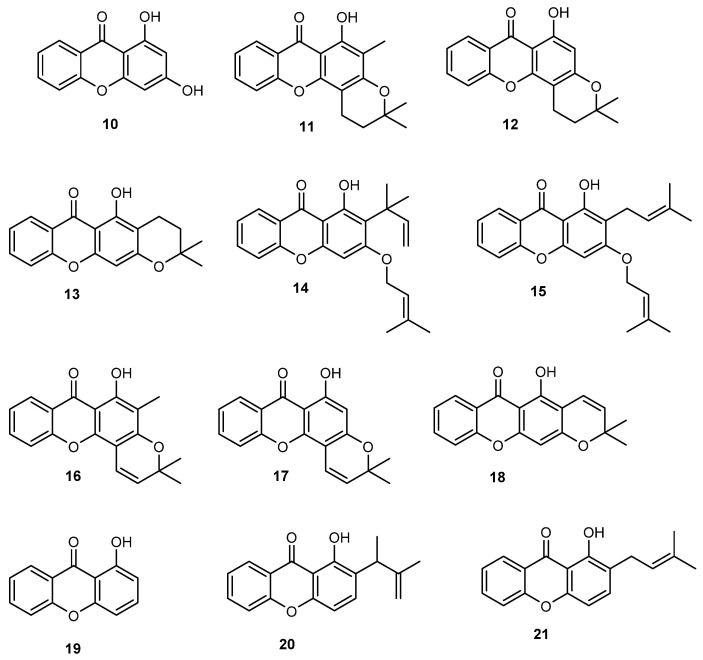
Synthetic prenylated xanthones with promising in vitro growth inhibitory effect in human tumor cell lines identified by our group, reported in [84,85].

The synthesis of a library of prenylated xanthones was shown to be fruitful with many of the synthesized compounds presenting encouraging antitumor activity in different cell lines. The first research work of our group concerning the synthesis of bioactive prenylated xanthones, comprised the synthesis of a small library of *O*-prenylated xanthones using 1,3-dihydroxy-2-methylxanthone (**3**, Figure 4) as building block, previously identified by us as a promising in vitro growth inhibitor of human tumor cell lines [48], as well as the *nor*-derivative 1,3-dihydroxyxanthone (**10**, Figure 6) [84]. Among synthesized compounds, xanthones **14** and **15** with isoprenyl and 1,1-dimethylallyl groups and xanthone **11** with a 2,2-dimethyldihydropyran group, showed improved in vitro growth inhibitory activity against the breast cancer MCF-7 cell line, if compared with their parent compounds **3** and **10**, while the growth inhibitory activity against the other cell lines was lost, suggesting some selectivity to MCF-7 cells [84].

In 2009, a rigidification strategy aiming to improve the antitumor activity of previously synthesized dihydropyranoxanthone derivative **13** was applied by our group and pyranoxanthones **16**–**18** were obtained by unsaturation strategy applied to the dihydropyran ring of dihydropyranoxanthones **11**–**13**, respectively (Figure 6) [85]. Moreover, two new *C*-prenylated derivatives (**20** and **21**) were synthesized by prenylation of xanthone **19** (Figure 6). Interestingly, when comparing the effects of the pyranoxanthones **16**–**18** on the growth of MCF-7 cells with those of their respective dihydropyran xanthones used as building blocks **11**–**13**, it was found that the presence of the unsaturation in the pyran ring was associated with loss of inhibitory activity against MCF-7, suggesting that the rigidification failed to give an improvement of activity. However, *C*-prenylation of the inactive xanthone **19** was associated with the appearance of some growth inhibitory effect against MCF-7 of derivatives **20** and **21** [85].

**Figure 7:**
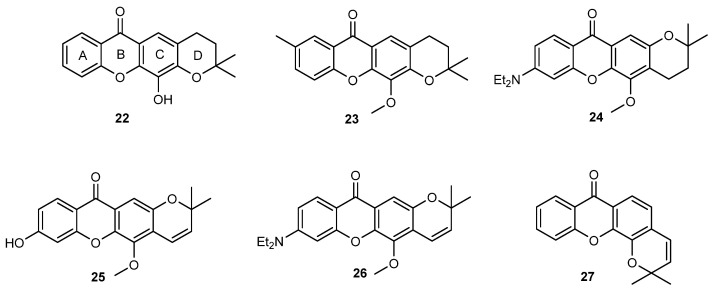
Xanthones with improved growth inhibitory activity against MCF-7, NCI-H460, A375-C5 and HL-60 cell lines reported in [78,80].

Aiming to pursue the study of the influence of prenylation in the antitumor activity of xanthones, in 2010 the synthesis of both open-chain and fused prenylated derivatives of 3,4-dihydroxyxanthone (**9**, Figure 4) was accomplished [86]. All the synthesized compounds reduced cell viability of K-562 cell line, derived from a blastic phase of human chronic myelogenous leukemia, using a trypan blue exclusion assay, being 12-hydroxy-2,2-dimethyl-3,4-dihydro-2*H*,6*H*-pyrano [3,2-*b*]xanthen-6-one (**22**, Figure 7) the most potent compound. Additionally, **22** decreased cellular proliferation, induced S-phase cell cycle arrest and apoptosis, being this effect associated with an increase of cleaved PARP and Bid, as well as a decrease in Bcl-xL in K-562 cells. Further studies in K-562 leukemia cells also revealed that **22** can act as a non-competitive P-gp inhibitor, pointing out its potential to act in MDR cancer cells [66]. Additionally, **22** showed promising in vitro growth inhibitory effect in estrogen-dependent ER (+) MCF-7 (breast) cells [87]. Interestingly, an enhancement in the antiestrogenic effect of 4-hydroxytamoxifen was also observed in ER (+) MCF-7 cells treated with the xanthone **22** [87]. Considering that the pyranoxanthone **22** was shown to exhibit potent antiproliferative activity in leukemia [86] and breast cell lines [86,87], this hit compound was selected for further investigation concerning the mechanism of action at a molecular level and the optimization of potency through chemical and nanotechnology approaches.

Considering the disclosed mechanism elucidation for naturally occurring prenylated xanthone α-mangostin and gambogic acid as inhibitors of MDM2-p53 interaction by some of us [88] and the promising growth inhibitory activity of **22** in cancer cell lines [86,87], this pyranoxanthone as well as other structure related compounds were submitted to in silico and in vitro studies in order to explore their potential as novel MDM2-p53 inhibitors. The p53 tumor suppressor is a major transcription factor with a crucial role in cell proliferation and death. The activity of p53 is commonly lost in cancers either by mutation in the TP53 gene, or by inactivation due to the overexpression of the main endogenous negative regulator, murine double minute 2 (MDM2). Therefore, restoration of p53 activity by inhibiting the MDM2-p53 interaction represents an appealing therapeutic strategy for many wild-type p53 tumors with overexpressed MDM2 [89]. Compound **22** was in fact identified as a putative inhibitor of MDM2-p53 interaction using a yeast phenotypic assay for the screening of inhibitors of MDM2-p53 interaction in association with a yeast p53 transactivation assay [90]. The activity of **22** as inhibitor of MDM2-p53 interaction was further validated in human tumor cells expressing wild-type p53 and overexpressed MDM2. Remarkably, **22** mimicked the activity of known p53 activators, leading to p53 stabilization and activation of p53-dependent transcriptional activity. In addition, it increased p21 and Bax protein levels, and caspase-7 cleavage. Computational docking studies allowed predicting that, like nutlin-3a, a known small-molecule inhibitor of MDM2-p53 interaction, **22** binds to the p53-binding site of MDM2. With this research line, a novel small-molecule inhibitor of MDM2-p53 interaction with a xanthone scaffold was identified for the first time, which could be used as molecular probe [90] and inspired other researchers to develop new anticancer agents targeting p53 [91]. Nevertheless, using adequate software (ACD/Labs software, Toronto, Canada) the calculated maximum water solubility of **22** was only 16 µg/mL [92]. This poor aqueous solubility could be a major drawback for its potential use in therapy. Therefore, drug delivery systems, including nanosphere, nanocapsule and nanoparticle formulations were developed [92,93]. Among these, nanocapsules containing poly(D,L-lactide-co-glycolide) (PLGA), polyvinyl alcohol (PVA) and Mygliol^®^ 812 were found to be noncytotoxic to MCF-7 cell line and **22**-loaded nanocapsules with an incorporation efficiency of 77% revealed to be more potent than the free compound against cell growth inhibition, which may be related to the enhancement in its intracellular delivery [90]. These results suggest that it was possible to enhance the effect of the hit compound **22** through the development of suitable noncytotoxic polymeric nanoparticles. In addition to nanotechnological approaches to optimize **22** previously described, our group has also been involved in the optimization of the potency of this xanthone through a chemical approach. Therefore, **22** was used as the starting point in the search for more potent antitumor agents. To increase the probability of success, the pharmacokinetic behaviour was also considered in this research plan. Accordingly, it was decided to optimize this compound following a multidimensional approach regarding, in parallel, at the activity and physicochemical properties, with the latter being used as a tool to predict the pharmacokinetic behaviour.

**Scheme 4:**
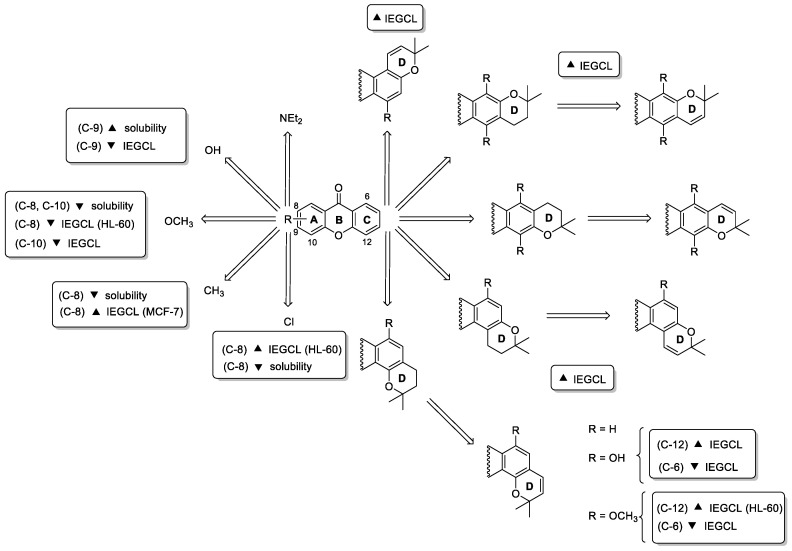
Putative SAR and structure–solubility relationship considerations for the analogues of **22** reported in [78,80] (IEGCL: inhibitory effect in growth of tumor cell lines).

A first series of 17 analogues of **22** with a linear tetracyclic system and different substituents in ring A were synthesized and evaluated for their antiproliferative activity against MCF-7 (breast adenocarcinoma), NCI-H460 (non-small cell lung cancer), A375-C5 (melanoma) and HL-60 (acute myeloid leukemia) cell lines. A new route, through a benzophenone intermediate was developed for the synthesis of **22** and its 17 analogues bearing different substituents on ring A and different ring D orientation. Some of the newly synthesized compounds (**23**–**26**) were more active than **22** in the four tumor cell lines tested (Figure 7). Particularly, analogue **24** with an 8-diethylamino group at A ring and a 2,2-dimethyl-3,4-dihydropyrano ring linked to the B ring of xanthone nucleus showed to be the most active in all tested cell lines. In general, like **22**, all synthesized analogues were more active towards the HL-60 tumor cell line, with the most potent compound (**23**) having a GI_50_ of 5.14 µM, lower than the hit compound **22** (GI_50_ of 23.41 µM). Interestingly, all compounds showed a logKp between 3 and 5 in two membrane models, namely liposomes and micelles, and all tested compounds showed low solubility, being this explained by their high rigidity and planarity [80]. Overall results allowed concluding some considerations concerning structure-activity and structure-solubility relationships, which are highlighted in Scheme 4.

A second series of 20 analogues of **22** with linear and angular fused pyran and dihydropyran rings were also synthesized and evaluated for their lipophilicity and antiproliferative activity in human tumor cell lines in order to perform SAR studies concerning the effect of the fused ring orientation and oxygenation pattern in pyranoxanthones (Scheme 4) [78]. The compounds were synthesized either by molecular modification of simple oxygenated xanthones or by total synthesis through benzophenone and diaryl ether routes. The introduction of pyran rings in simple oxygenated xanthones were accomplished either by the cyclization with platinum of the dimethylpropargyl aryl ethers, or by the condensation with prenal or by the reaction with prenyl bromide catalysed by Montmorillonite K-10 and MW heating. The total synthesis of pyranoxanthones was carried out via diaryl ether and benzophenone using the appropriate benzopyrans and carboxylic acid derivatives as building blocks. From this work, the angular pyranoxanthone **27** (Figure 7) emerged as the most potent cell growth inhibitor of tested human tumor cell lines (3.2 < GI_50_ < 13.3 µM), showing suitable drug-like lipophilicity. Overall results obtained for the evaluation of the cell growth inhibitory activity in the referred four tumor cell lines allowed to advance some putative SAR summarized in Scheme 4.

In 2020, to further explore the mode of action of **22** in cells, as well as other structure related(thio)xanthones with antitumor activity, the transcriptional response of the filamentous fungus *Neurospora crassa* to these compounds using high throughput RNA sequencing was evaluated. These compounds showed to induce genes which express ABC transporters in *N. crassa*, particularly atrb and cdr4, and to repress genes that are evocative of genes downregulated during oxidative stress [94].

Although the antitumor activity of prenylated xanthones was the most reported biological activity, the potential of these compounds as AF agents has recently been explored by our group. In this context, and as a part of our efforts to discover innovative antifoulants inspired in natural products, xanthone **22** was also investigated along with the simple oxygenated xanthones (Section 2.1). This prenylated xanthone **22** showed even more potent in vivo activity toward the settlement of *Mytilus galloprovincialis* larvae (EC_50_ = 4.60 µM) than the dihydroxyxanthone **9**, showing also a low toxicity profile to this macrofouling species (LC_50_ > 500 µM and LC_50_/EC_50_ > 108.70) and to *Artemia salina* (<10% mortality at 50 µM). Concerning the mechanisms of action in mussel larvae, xanthone **22** showed a specific target directly related with the proximal thread proteins (TMPs), which are expressed by bivalve molluscs that adhere to underwater surfaces through the production of byssal threads [55].

To sum up, 50 new prenylated xanthones were synthesized by our group using classical and non-classical methodologies. Notably, MAOS and the combination of heterogeneous catalysis (K10 clay) with MW irradiation were described for the first time for the synthesis of prenylated xanthones. The majority of prenylated derivatives were studied for their in vitro growth inhibitory activity against tumor cell lines. The pyranoxanthone **22** emerged as the most promising in vitro growth inhibitor, being this effect associated mostly with a MDM2-p53 inhibitory effect. Nevertheless, other 26 compounds had also good outcomes against tumor cell lines; the results obtained allowed us to establish structure-activity and structure-solubility relationship considerations which will allow in the future the design of new prenylated xanthones with improved antitumor activity.

### 2.3. Halogenated and Carboxyxanthones

**Figure 8:**
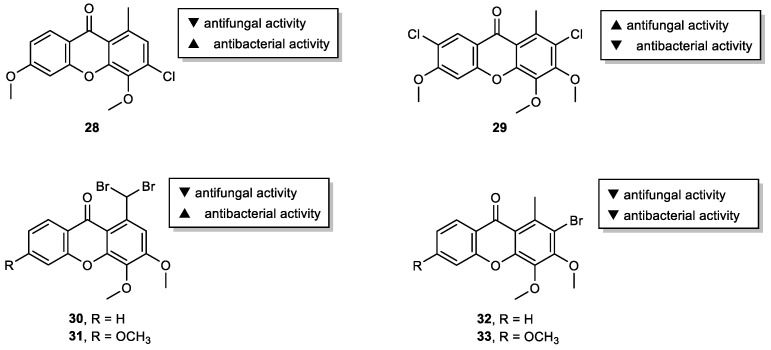
Antimicrobial halogenated xanthones **28**–**33**.

Over the preceding decade, more than 100 xanthones from lichen sources were identified [95], but only a limited number have been investigated for their bioactivities [31]. Particularly, chlorinated lichen xanthones have been found attractive for their antibacterial and antifungal activities [96]. Based on these models, a series of novel chlorinated xanthones with different substitution patterns were prepared and evaluated for its antimicrobial activity potential [29]. The presence of chlorine at C-3 seems to have some influence on the antibacterial activity since 3-chloro-4,6-dimethoxy-1-methyl-9*H*-xanthen-9-one (**28**) showed promising antibacterial activity against *E. faecalis* (ATCC 29212) and *S. aureus* ATCC 29,213 (Figure 8). On the other hand, 2,7-dichloro-3,4,6-trimethoxy-1-methyl-9*H*-xanthen-9-one (**29**), with chlorine atoms at C-2 and C-7, exhibited potent antifungal activity suggesting that their joint presence may be required for this effect, similarly to the natural xanthone, thiophanic acid. Inspired by these results and in order to further extend the diversity of the library, our group also explored the potential of brominated xanthones as antimicrobial agents [53]. Although no antifungal activity was observed for the tested compounds, 1-(dibromomethyl)-3,4-dimethoxy-9*H*-xanthen-9-one (**30**) and 1-(dibromomethyl)-3,4,6-trimethoxy-9*H*-xanthen-9-one (**31**) exhibited antibacterial activity against all seven bacterial (including two multidrug-resistant) tested strains (Figure 8). While the studied xanthones exhibited great potential as antibacterial (**28**, **30** and **31**) or antifungal (**29**) agents, the low solubility displayed by some derivatives limited further screenings. Nevertheless, these can be used in the future as models to improve drug-like properties. In the scope of the same study, other xanthone derivatives modified at C-1 (CH_3_, CHO, COOH, COOCH_3_, or NOH) were also synthesized and screened for their antimicrobial activity. However, none of the tested compounds showed relevant activity against the five fungal and seven bacterial (including two multidrug-resistant) strains tested.

**Figure 9:**
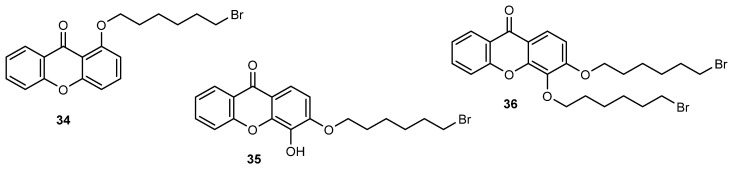
Bromohexyloxyxanthones **34**–**36**.

In a study involving the synthesis of dimers of xanthones as bis-intercalators [97], bromohexyloxyxanthones **34**–**36** were obtained as side products and were investigated for their effect on the in vitro growth of human tumor cell lines (Figure 9). Although for bromoalkoxyxanthone **35** no relevant growth inhibition was observed on all the tested tumor cell lines, the bromoalkoxyxanthones **34** and **36** showed an interesting growth inhibitory effect, higher than the parent hydroxylated xanthones and the target bisxanthones. Although more studies are needed to establish a SAR, results showed that one free hydroxyl group in the bromoalkoxyxanthone scaffold **35** is unfavourable to the growth inhibitory activity. Additionally, X-ray crystallography allowed the structure elucidation of 1-(6-bromohexyloxy)-xanthone (**34**) which can be helpful in the future to disclose some mechanistic aspects concerning the binding of **34** to DNA or other putative targets.

**Figure 10:**
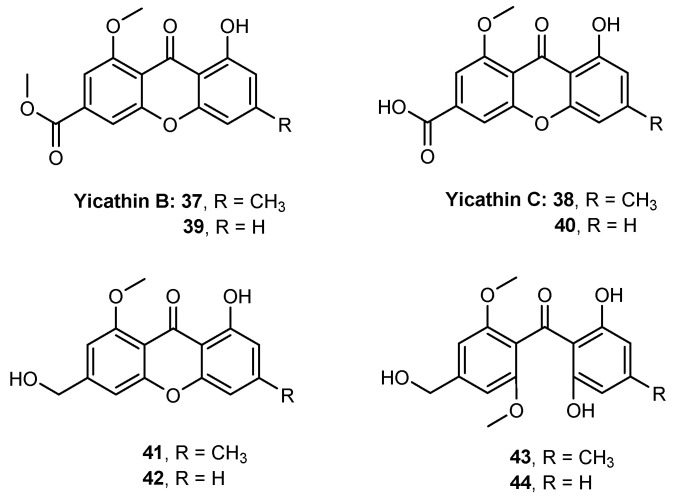
Structure of yicathin B (**37**) and yicathin C (**38**) and their six analogues **39**–**44**.

Yicathins B and C are two marine natural xanthones that have shown antibacterial and antifungal activities [98]. The total synthesis of yicathin B (**37**) and yicathin C (**38**), along with six analogues (**39**–**44**) was reported by our group (Figure 10) [73]. Lipophilicity of yicathins and their analogues was evaluated using in silico and experimental biomimetic methodologies. The obtained partition coefficients were quite different, namely for ionized compounds at physiological pH. Nevertheless, the lipophilicity of the synthesized compounds were within the limits preconized by the most common “drug-like” guidelines. In vitro antitumor and anti-inflammatory activities were evaluated. Antitumor activity was screened in three human tumor cell lines, but the compounds did not reveal a significant ability to inhibit cell growth, being 48.70 μM the lowest GI_50_ value. Nevertheless, compounds **37**, **41**, and **43** showed a significant in vitro anti-inflammatory activity, which was comparable with well-known nonsteroidal anti-inflammatory drugs (NSAIDs), like diclofenac and celecoxib.

### 2.4. Aminated Xanthones

**Figure 11:**
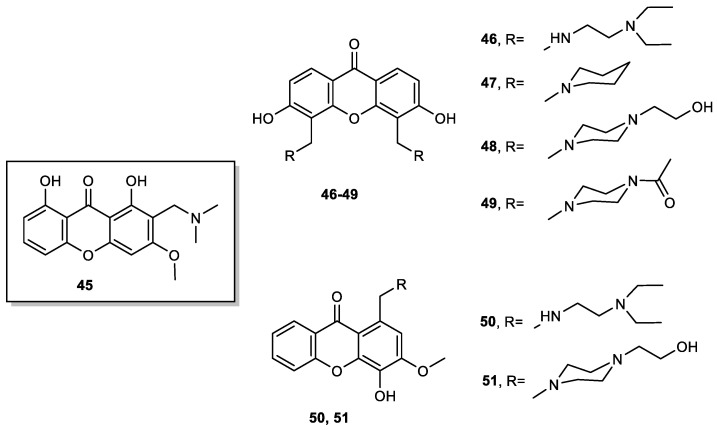
Aminated xanthones **45**–**51**.

Our group performed a complete SAR study through the analysis of various xanthone derivatives with multitarget activity against Alzheimer Disease (AD) [99]. It was found that the presence of a methoxy group at C-3 increased the inhibition of MAO as well as the inhibition of acetylcholinesterase (AChE) and the presence of a hydroxyl group on the xanthone scaffold was important for antioxidant activity. Based on this study and considering the potential of tricyclic amine drugs for CNS diseases, our group designed and prepared several xanthone derivatives to be used as dual agents for AD with AChE inhibitory and antioxidant activities [100]. Aminated xanthone **45** was obtained from 1,8-dihydroxy-3-methoxy-9*H*-xanthen-9-one by Mannich reaction with formaldehyde and dimethylamine (Figure 11), while xanthones **46**–**51** were obtained by reductive amination from the corresponding carbaldehydes (Figure 11), previously obtained from oxygenated xanthones by a Duff formylation. From this study, xanthone **45** emerged as a hit compound exhibiting interesting dual AChE inhibitory effect and antioxidant activity (ferrous and copper ions chelating properties).

**Figure 12:**
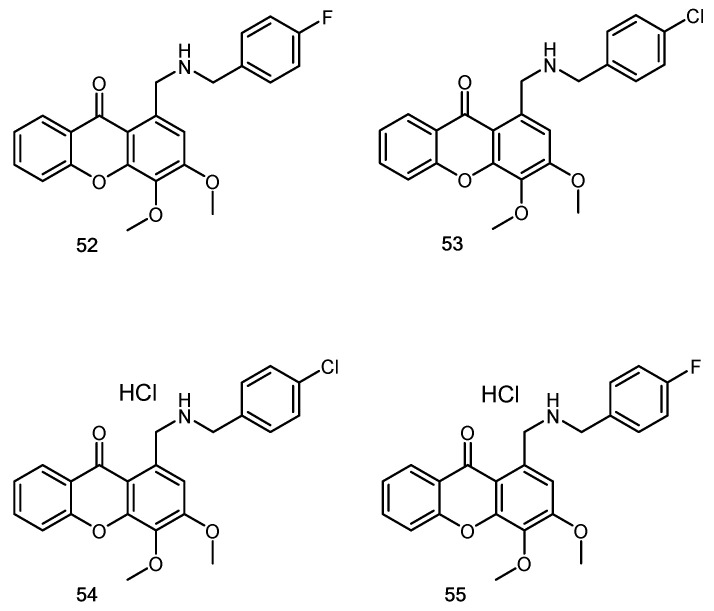
Aminated xanthones **52**–**55**.

Another example of hybridization approach was also explored by us regarding the potential of aminated xanthones to disrupt the MDM2-p53 interaction using an yeast cell-based assay [101]. Previous studies developed by our group disclosed two xanthone derivatives, pyranoxanthone **22** (Figure 7) and 1-carbaldehyde-3,4-dimethoxyxanthone **7** (Figure 4) as MDM2-p53/TAp73 inhibitors. Hence, xanthone **7** was used as starting material for the synthesis of a series of eleven aminated xanthones via reductive amination. From this study, xanthone **52** (Figure 12) was identified as a putative p53-activating agent, inhibiting the growth of human colon adenocarcinoma HCT116 cell line, being this effect associated with cell cycle arrest through activation of the p53 pathway. Although further studies are required to confirm the mechanism of action of **52**, these results demonstrated the potential usefulness of coupling amine-containing structural motifs of known MDM2-p53 disruptors into the 3,4-dioxygenated xanthone scaffold, which may lead to the identification of a novel xanthone derivative with promising antitumor activity.

Later, another series of eleven aminated xanthones, together with xanthone **7** (Figure 4), 1-(hydroxymethyl)-3,4-dimethoxy-9*H*-xanthen-9-one and the aminated xanthone **52** previously studied for their ability to disrupt the MDM2-p53 interaction, were also screened for their antimicrobial activity against five fungal and seven bacterial (including two multidrug-resistant) strains [53]. The results of antimicrobial screening revealed the potential of compounds with halogen atoms as the most promising in terms of antibacterial activity, with some of the representatives (**53**, **54** and **55**) being highly active against either Gram-positive or Gram-negative strains. Regarding to antifungal activity, compound **54** revealed a potent inhibitory effect on the growth of dermatophyte clinical strains (*T. rubrum* FF5, *M. canis* FF1 and *E. floccosum* FF9). The fungicidal activity was observed for compound **55** with *T. rubrum* FF5. Also, **54** showed a potent inhibitory effect of *C. albicans* ATCC 10,231 germ tube and biofilm formation, important virulence factors. Due to low solubility, conversion to the corresponding hydrochloride salts increased the potential of the studied aminated xanthones as antibacterial or antifungal agents [53].

**Figure 13:**
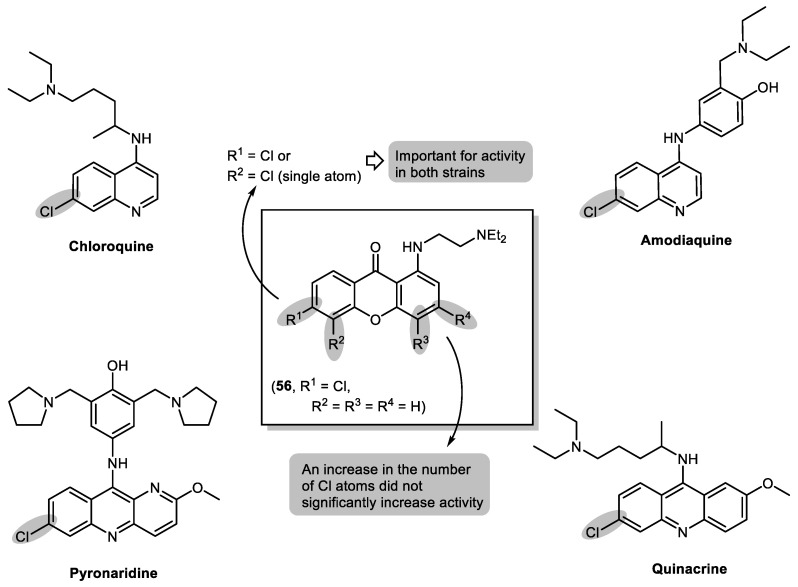
Structures of known antimalarial drugs based on chlorinated 4-aminoquinolines and putative SAR analysis of aminated xanthones and hit compound **56**.

Hydroxylated xanthones have previously revealed to have antimalarial activity, pointing to the same molecular mechanism as antimalarial 4-aminoquinolines mefloquine, halofantrine, and quinine. [102] This mechanism consists in preventing the process of haemozoin formation; hence, the consequent concentration of free haematin would be sufficient to kill the parasite [102]. These compounds might interact with haematin in the form of a µ-oxo dimer or a dimer of haemozoin. This interaction would lead to stabilization of haematin in a soluble form, consequently leading to parasite death [103]. Aided by docking simulations [103], our group designed a series of twelve new chlorinated 9*H*-xanthones with a [2-(diethylamino)ethyl]amino group in position C-1 and one to four Cl-atoms in different positions (Figure 13), and studied their antimalarial activities [104,105]. All twelve compounds were found to be active against the chloroquine-susceptible and chloroquine-resistant strains 3D7 and Dd2, respectively, of *Plasmodium falciparum*. A more pronounced effect was observed for strain 3D7 (IC_50_ = 1.7–18.1 µM) relative to strain Dd2 (IC_50_ = 3.9–66.4 µM). From this study, compound **56** (Figure 13) emerged as a hit compound due to significant in vitro activity (IC_50_ = 3.9 µM) towards the resistant Dd2 strain. The position and number of the Cl substituents are of great importance regarding activity. When compared to known antimalarial drugs, a Cl atom in position C-6 seems to be important for activity in both strains, as well as a single Cl atom in position C-5, indicating a similar mechanism of action.

Due to their versatility, aminated xanthones have been widely studied over the last years by our research group. The main limitation of these compounds is their low solubility in water, which was easily overcome through the conversion into hydrochlorides. The synthesis of hydrochloride salts also enhanced their activity (which was validated in the antimicrobial screening).

### 2.5. Sulfated and/or Glycosylated Xanthones

**Figure 14:**
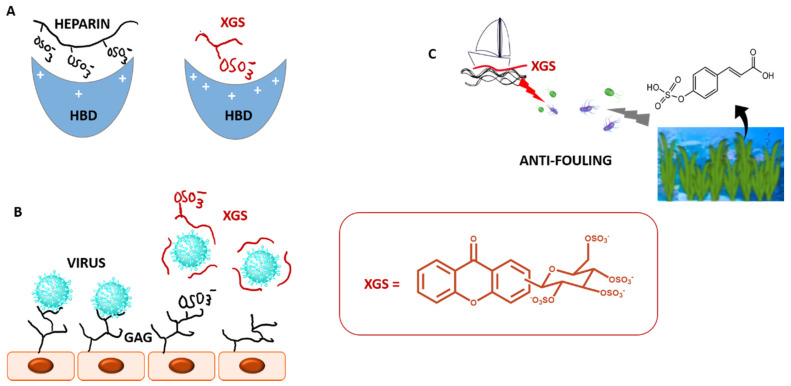
Sulfated and glycosylated xanthones (XGS) as mimetics of several physiologic processes: (**A**). Anticoagulant effects as mimetics of heparin, (**B**). Antiviral effects as mimetics of GAGs; (**C**). Antifouling effects as mimetics of marine antifouling sulfated metabolites. HBD: heparin binding domain; GAG: glycosaminoglycan.

Heparin polysaccharide is decorated by numerous ionic groups, via sulfate and carboxylate groups. Since heparin has an average molecular weight of 15 kDa, this property gives heparin the highest negative charge density of any known naturally derived biomolecule. Although heparin has been one of the most effective and widely used drugs of the past century [106], it is beset with many complications including haemorrhage, thrombocytopenia, osteoporosis and inconsistent patient response, owing to its polyanionic, polymeric and polydisperse nature. Functional mimics of heparin without its adverse effects are highly desirable as an alternative for anticoagulant therapy [107]. Replacing the sulfate groups in the binding domain of heparin with other anions nullifies its anticoagulant activity. Therefore, sulfated small molecules are expected to preserve some molecular properties of heparin but with reduced anionic character, higher hydrophobic nature, and feasible synthesis (Figure 14A).

**Figure 15:**
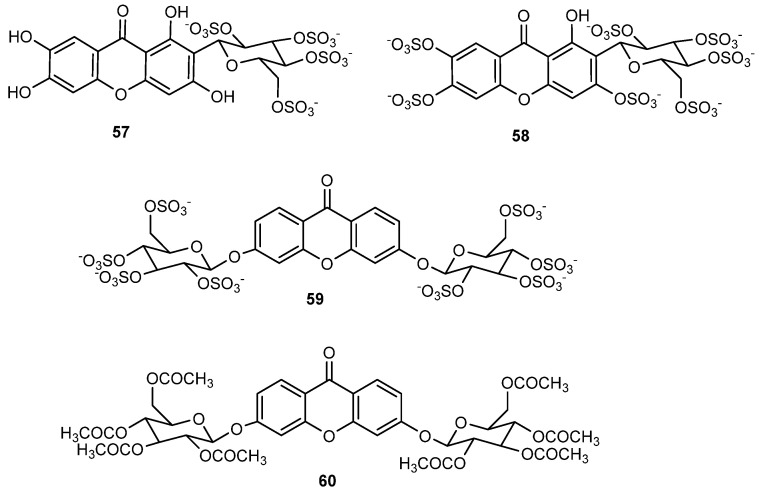
Sulfated and/or glycosylated bioactive xanthones.

In this direction, two polysulfated derivatives of the naturally occurring glycosylated xanthone, mangiferin, were prepared by our group (**57** and **58**, Figure 15) to study their anticoagulant effects [108].

Mangiferin was selected for sulfation as part of our top priority of developing new molecules derived by molecular modifications of known drugs, for several reasons as follows. Modification of molecules that have already been used in human therapy leads to compounds with more predictable and less complex pharmacokinetics, lower incidence of side effects, and less demanding clinical studies. The wide use of mangiferin by humans for many years suggests low toxicity not only for mangiferin but also for their metabolites and analogues. Protective effects of mangiferin with regard to cardio, renal, liver, and brain toxicity are well documented [109]. Because aryl *C*-glycosides are stable analogues of *O*-glycosides, resistant to metabolic processes, the *C*-glycoside mangiferin emerged as an interesting derivative to be sulfated. Starting from commercial mangiferin, sulfation was performed with triethylamine sulfur trioxide adduct in dimethylacetamide at 65 °C for 24 h. Depending on the loading of sulfur trioxide adduct, mangiferin tetrasulfate (**57**) and mangiferin heptasulfate (**58**) were obtained [108]. Due to the sulfate groups, **57** and **58** revealed a better solubility in water than mangiferin. Both derivatives exhibited anticoagulant activity when tested in vitro in human plasma, with the heptasulfate mangiferin (**58**) being more potent. Enzymatic in vitro assays to elucidate coagulation targets of **58** demonstrated that this sulfated mangiferin was a selective direct inhibitor of factor Xa. This work inspired Q. Rashid et al. to explore by in silico studies the binding mode of several sulfated and non-sulfated xanthones on coagulation proteases [110].

A xanthone *O*-glycosylated on both rings (**59**, Figure 15) was also planned to interact in an extended binding area of proteins of the coagulation process [111]. According to this hypothesis, the sulfated diglycosylxanthone would lead to a molecule with higher molecular size and number of sulfate groups than the previous polysulfated mangiferin derivatives and thus increase anticoagulant effects. As planned, this octasulfated xanthone was more potent than the tetra and heptasulfated mangiferins and was shown to activate coagulation by a dual mechanism, inhibiting the factor Xa directly and via antithrombin III activation [108].

In addition to anticoagulant effect, xanthonosides **58** and **59** were also endowed with antiplatelet effects. Their antiplatelet activity was attributed to the inhibition of arachidonic acid and ADP-induced platelet aggregation [108]. The dual antiaggregant behaviour of these small sulfated molecules affords an opportunity to discover different molecules from the known antiplatelet agents with possibly different pharmacological profile. Dual antiplatelet therapy with acetyl salicylic acid (inhibitor of arachidonic acid-induced platelet aggregation) and a thienopyridine (inhibitor of ADP-induced platelet aggregation) is often recommended for high-risk patients. On the other hand, molecules such as xanthones **58** and **59**, which simultaneously target the antiplatelet and anticoagulant pathways, could be useful to prevent and treat both venous and arterial thrombosis. In the past decade, dual anticoagulant/antiplatelet agents have been persuaded, although such dual agents have not yet achieved clinical development [112].

A putative in vivo anticoagulant efficacy in mice, with no suggestion of acute side effects, shows the potential of xanthones **58** and **59** to lead a new class of therapeutic agents in the prevention and treatment of thromboembolic events. These scaffolds with lesser charge density compared to heparins may be expected to cause minimal cross-reactivity with other proteins. Therefore, these compounds are likely to overcome the bleeding complications and hepatic toxicity owing to their minimal charge density. This family of compounds, with a well-defined composition, are also merited with a feasible synthesis protocol [107,113].

A number of viruses use sites on heparan sulfate (HS) as receptors for binding to cells [114]. Mimics of these sequences are likely to interact with the virus in solution, thereby tying it up and disrupting the viruses interaction with cell surface HS (Figure 14B) [115]. Therefore, the potential of our sulfated small molecules as antivirals was later explored by our group. It was discovery that sulfated xanthones **58** and **59** were able of decreasing Epstein–Barr virus (EBV) levels in a Burkitt lymphoma (BL) [116]. EBV infects more than 90% of the world population. Following primary infection, EBV persists in an asymptomatic latent state, associated with several diseases, such as BL. To date, there are no available drugs to target latent EBV, and the existing broad-spectrum antiviral drugs are mainly active against lytic viral infection. The fact that compounds **58** and **59** led to a decrease in the EBV DNA present in the Raji cell line (Burkitt lymphoma) was a first indication that these molecules were targeting EBV in its latent state or interfering with episome maintenance. The use of hydroxyurea (an anticancer drug) to reduce EBV DNA load, although having been successful, has the disadvantage of leading to the accumulation of additional mutations in the cellular genome. In this context, the sulfated xanthones revealed by our group may serve as a starting point for development of alternative molecules to hydroxyurea. In addition, these compounds did not present cytotoxicity at the concentrations tested for their antiviral activity what is relevant for their possible use as antivirals, being potentially specific and not affecting cell viability.

Nature uses sulfation mainly to avoid potential toxicity, so synthesis of non-natural sulfated small molecules could be a way in generating new nontoxic bioactive agents. Zosteric acid, which is a *p*-sulfated cinnamic acid derived from the marine seagrass *Zostera marina*, has received much attention as a potential marine antifoulant product (Figure 14C) [117]. The toxicity studies of zosteric acid showed no measurable LD_50_ for larval fish and an acute toxicity profile similar to table sugar. The marine industry is now facing the phase-out of current persistent, bioaccumulative, and toxic biocides that are continuing being released from AF coatings to the environmental, shortening the available alternatives to combat marine biofouling. Developing alternative environmentally friendly and non-toxic AF agents is an urgent demand. The yields of natural compounds from marine organisms are generally poor, hindering their development as AF agents. Moreover, optimizing a microorganism for enhanced production of antifoulant is generally laborious and time consuming. Besides, marine natural products are often structurally too complex to be synthesized on a large scale and are often not compatible with polymer coatings. Synthesis of simple and optimized antifoulants looks like a more sustainable way bringing an opportunity to produce commercial supplies for AF industry without sacrifice natural species. Therefore, in 2017, inspired by the marine zosteric acid molecule, several synthetic sulfated structure-diverse compounds obtained by our group were tested for AF properties [118]. New synthetic AF scaffolds were disclosed in this work, namely xanthone **59**, which exhibited effective anti-settlement response towards the most common biofouling species with worldwide representatives, the *Mytilus galloprovincialis* (EC_50_ = 23.19 μM) without causing mortality to this species at any of the tested concentrations (LC_50_ > 500 μM), in contrast to the commercial eco-friendly AF agent ECONEA^®^ which showed some toxicity at the higher concentrations tested (LC_50_ = 107.78 μM). For the efficacy level of new eco-friendly alternatives, it is highly recommended therapeutic ratios (LC_50_/EC_50_) higher than 15, which is in accordance with the results obtained with this new AF agent. However, while this xanthone **59** was active, other sulfated xanthones did not show notable activity. These results show that the nature of the scaffold plays a role in placing sulfate groups in favourable position for the activity: while xanthone **59** was highly active, a structure related xanthone in which the *O*-linkage was replaced by a triazole linkage, was not active.

In contrast to the biocide ECONEA, which caused 100% mortality of the marine shrimp *Artemia salina* at 50 µM, compound **59** was not significantly different from filtered seawater, revealing no toxicity even at 250 μM. The nontoxic nature of xanthone **59** was also confirmed by the lack of light radiation inhibition on the *Vibrio fischeri* luminescent assay (LC_50_ > 1000 μg/mL). Adding to the low ecotoxicity potential, the bioactive sulfated xanthone is highly water soluble, suggesting also a low bioaccumulation potential, which was proven by in silico calculations of logKow (octanol-water partition coefficient). In conclusion, the first synthetic xanthone with AF effects was disclosed by our group in 2017 [118], merited with putative eco-friendly label. The viable synthetic process can predict its easy scale-up and future commercialization. This work was the starting point for the development of new optimized and/or diverse xanthones with AF activity (Section 2.1 and Section 2.2) [55].

Natural glycosides of xanthones demonstrate several biological activities in which the glycosidic moiety showed an important role [18]. In our group, an acetylated xanthone glycoside (**60**, Figure 15) obtained by synthesis was recently shown to have a potent inhibitory effect on glioma cells growth [119]. Acetylated xanthonoside **60** and its parent non-acetylated xanthonoside were first evaluated for their in vitro growth inhibitory effect on A375-C5 (IL-1 insensitive malignant melanoma), MCF-7 (breast adenocarcinoma), and NCI-H460 (non-small-cell lung cancer) cell lines. Acetylated xanthonoside **60** exhibited potent growth inhibitory activity in MCF-7 and NCI-H460 cell lines with GI_50_ values of 0.46 and 0.19 μM, respectively, while the non-acetylated xanthonoside was not able to reach 50% of cell growth inhibition. These results put forward the role of the acetyl groups in the potent cell growth inhibitory activity of xanthone **60**. After this promising results, glioblastoma cell lines (U251, U373, and U87MG) growth was also studied in the presence of xanthone **60** and a potent inhibitory activity was found with this xanthone against the referred three lines (GI_50_ values between 0.42–0.55 μM). Association of acetyl groups with inhibition of growth of glioma cell lines had been reported in the natural *O*-acetylated ganglioside GD1b neurostatin, present in the mammalian brain, which showed strong inhibition of astroblast and astrocytoma division and in a previous work of our regarding an acetylated flavonoid glycoside [120]. Acylation is expected to increase the penetration through the cell membrane. However, as acetylated compounds are likely to present poor water solubility and to suffer hydrolysis by esterases, the encapsulation of this acetylated xanthonoside in nanosystems was planned as possible strategy to overcome these limitations. Xanthone **60** was successfully incorporated by us in liposomes while maintaining the inhibitory activity against glioblastoma cell lines [119]. New formulations and/or drug carriers to fight glioma growth with this promising xanthone are being studied by our group.

### 2.6. Chiral Derivatives of Xanthones

The relationship between chirality and biological activity has been of increasing importance in medicinal chemistry [121], and our group has contributed to this research field with the synthesis of a library of chiral derivatives of xanthones (CDXs). Our inspiration to follow this way was based on Nature, as an unquestionable successful source of chiral bioactive compounds [122]. Actually, in biological systems, chirality is the rule rather than the exception and the presence of chiral centers in natural products, provides higher affinity and target specificity. Regarding this class of compounds, many naturally occurring xanthones, isolated from terrestrial and marine sources are chiral, and present a wide range of biological and pharmacological activities [31]. Nevertheless, while biosynthetic pathways frequently give only one enantiomer, the synthetic procedures may allow the preparation of both enantiomers, in addition to the access to structures that otherwise could not be reached [16,123]. Therefore, our main goals were to synthetize bioactive CDXs inspired in natural molecules and/or introduction of chirality in some interesting molecules, as well as obtaining both enantiomers with very high enantiomeric purity to explore enantioselectivity. We consider that it is of crucial importance to study the biological behaviour of each single enantiomer since the physiological properties, such as pharmacodynamics, pharmacokinetics and toxicity, are often different between enantiomers [124]. Moreover, enantioselectivity studies must be performed in the early stage of drug discovery and development. Another reason to work with CDXs was to enlarge chemical and biological spaces. In fact, despite the large structural multiplicity of bioactive xanthones, only a handful of synthetic chiral derivatives was reported [125,126]. Additionally, the CDXs were also obtained to achieve other purposes, such as to be used as analytical tools, as it will be covered at the end of this section.

The first CDXs synthesized by our group were inspired in natural molecules, specifically in xanthonolignoids. This natural class of compounds was chosen, as they are interesting bioactive and very promising templates for molecular modifications. They are isolated from plants of the Clusiaceae family (Guttiferae) and comprise a phenylpropane nucleus bounded to a xanthone scaffold by a dioxane moiety, formed by radical oxidative coupling [127]. Our group described, in 1987, the synthesis of *trans-*kielcorin (Figure 3) and its stereoisomer, *cis*-kielcorin, both xanthonolignoids of the kielcorin group [35]. It was the first report of the biomimetic synthesis of these compounds. While the total synthesis of kielcorin derivatives requires severe reaction conditions and many steps, the biomimetic pathway is based on natural building blocks and is achieved by an oxidative coupling of a suitable dihydroxyxanthone with a cinnamyl alcohol derivative, in the presence of an oxidizing agent at room temperature [127]. In 1999, also by a biomimetic approach, the synthesis of *trans*-kielcorin B (Figure 3) and *trans*-isokielcorin B was described by our group [128]. To obtain bioactive analogues with kielcorin scaffold, other constitutional isomers were synthesized by us in 2002, namely *trans*-kielcorin C (**61**), *trans*-kielcorin D (**62**), *trans*-isokielcorin D, *cis*-kielcorin C, and *trans*-kielcorin E [59]. Once again, the synthetic approach was performed on a biomimetic way, by oxidative coupling of coniferyl alcohol with an appropriate xanthone. The oxidizing agents Ag_2_O, Ag_2_CO_3_, K_3_[Fe(CN)_6_], among others, were used to investigate the oxidative coupling reactions [129].

**Figure 16:**
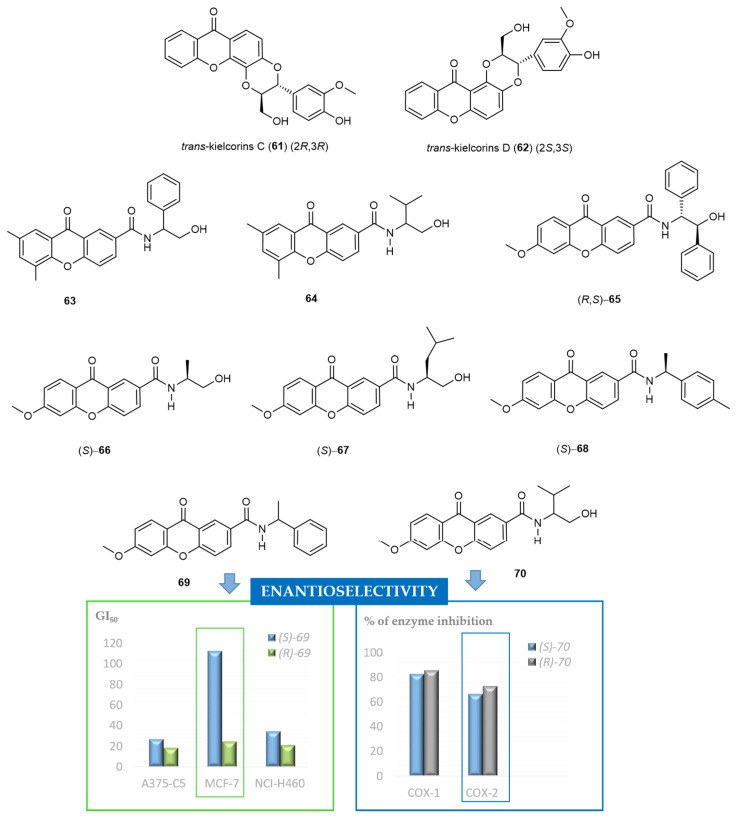
Chemical structures of representative bioactive CDXs and examples of enantioselectivity in biological activity.

Synthetic kielcorins C-E were evaluated for their in vitro effect on the growth of three human tumor cell lines, MCF-7 (breast), TK-10 (renal), UACC-62 (melanoma), and on the proliferation of human lymphocytes [62]. The growth inhibitory effect was moderate but in a dose-dependent manner and influenced by the isomerism. The most active compounds were *trans*-kielcorins C (**61**) and D (**62**) (Figure 16). The inhibition of human lymphocyte proliferation induced by phytohemagglutinin (PHA) was identified [56]. The high potency and selectivity observed for these compounds suggested that kielcorins might be an important model for developing potent and isoform-selective protein kinase C (PKC) inhibitors. Kielcorins C-E revealed an effect compatible with PKC inhibition similar to that exhibited by chelerythrine, a well-established PKC inhibitor [130]. Previously, synthetic *trans*-kielcorin (Figure 3) and *trans*-isokielcorin B were evaluated and demonstrated protective effect against *tert*-butylhydroperoxide-induced toxicity in freshly isolated rat hepatocytes [50], although in a less extension than their simple oxygenated precursors.

To determine the stereochemistry-activity relationship on the growth inhibitory effects of the kielcorins C-E, chiral liquid chromatography (cLC) methods were developed for their enantioseparation, using polysaccharide-based chiral stationary phases (CSPs) and multimodal elution conditions, in 2004 [131]. Later, the enantiomers of the kielcorins C-E were efficiently separated on a multimilligram scale using an amylose *tris*-3,5-dimethylphenylcarbamate CSP [132]. A solid-phase injection system was developed and combined with a closed loop recycling system to increase the productivity and recovery of the preparative process. The enantiomeric purity was also evaluated being higher than 99%, except for *trans-*kielcorin C (**61**) [132]. It must be highlighted that the development of methods to obtain and analyse both pure enantiomers has been acquired crucial importance, as are required for biological and pharmacological activity evaluation of both enantiomers [133]. The inhibitory activity of the racemates C-E as well as of the corresponding enantiomers on in vitro growth of the human breast adenocarcinoma cell line MCF-7 was evaluated and compared. The most evident enantioselectivity was found between the racemate of *trans*-kielcorin D (**62**) (inactive) and the active enantiomers (+)-**62** and (−)-**62** (Figure 16) [68,134,135,136].

More recently, in 2012, our group explored another approach to acquire synthetic CDXs in an enantiomerically pure form by binding diverse chiral moieties to an appropriate xanthone scaffold (Figure 16) [137]. Generally, the synthetic strategy used involved the coupling of suitable functionalized xanthones with both enantiomers of commercially available chiral building blocks, using a coupling reagent in the presence of a catalytic amount of triethylamine (TEA) and tetrahydrofuran as solvent. Through this “chiral pool” strategy, we currently have a large library of CDXs including eighty-six compounds described in literature [135,137].

**Figure 17:**
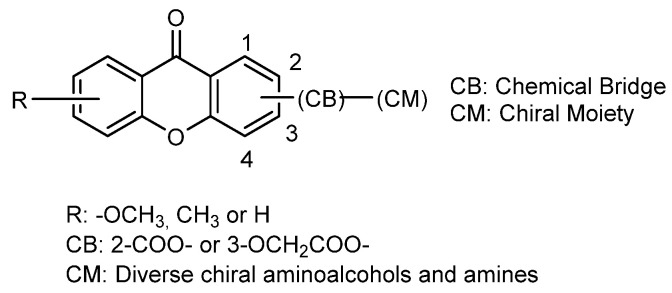
General structure of the CDXs with aminoalcohols and amines as CM.

Considering that carboxyxanthone derivatives are appropriate chemical substrates to perform molecular modifications to obtain diverse derivatives and analogues [138], we selected different compounds, comprising one or more carboxylic groups, to perform the referred syntheses [68,135,136,137]. Both enantiomers of a variety of amino alcohols, amines, and amino esters were chosen as chiral building blocks, containing a primary amine as reactive group for amide formation (Figure 17). Until now, *O*-(benzotriazol-1-yl)-*N*,*N*,*N*’,*N*’-tetramethyluronium tetrafluoroborate (TBTU) was demonstrated to be the best coupling reagent to synthesize CDXs, All products have no tendency towards racemization or enantiomeric interconversion. TBTU has been broadly used as an effective reagent for the synthesis of varied classes of compounds [139] however, our group reported for the first time the use of this coupled reagent to synthetize CDXs [137]. All coupling reactions were carried out at room temperature, showing short reactions times (ranging from 20 min to 6 h) and excellent yields (ranging from 92% to 99%) [135,136,137]. The synthetic methodology proved to be very efficient, with broad-scope applicability, and simple operationally. Moreover, it was found that the synthesis of the CDXs was easily scaled-up to obtain available quantities for biological and pharmacological assays as well as other applications [68,69].

Thirty-one enantiomeric pairs of the synthesized CDXs were evaluated for the in vitro growth inhibitory activity in three human tumor cell lines, A375-C5 (melanoma), MCF-7 (breast adenocarcinoma), and NCI-H460 (non-small cell lung cancer) [135,136]. The obtained results allowed to find active chiral compounds for all the tested cells lines, with some showing cell-type selectivity, and to draw SAR considerations. It was found that the nature and position of substituents on the xanthone scaffold and on the aromatic ring of the chiral moiety had a significant influence in determining the growth inhibitory effect. Another feature that has a decisive role on the growth inhibitory effect was the configuration of the stereogenic center(s). The most active CDXs in all human tumor cell lines were (*R*,*S*)-**65** [135] and (*R*)-**69** [136] (Figure 16). Both enantiomers of CDX **63** also revealed promising results [136] (Figure 16). The (*S*)-enantiomer of compound **69**, showed promising inhibitory activity against A375-C5 and NCI-H460 cell lines; however, it is important to point out the high enantioselectivity observed for MCF-7 tumor cell line with (*S*)-**69** showing low inhibitory effect (GI_50_ = 112.3 ± 19.56 μM) and (*R*)-**69** presenting a GI_50_ = 24.10 ± 8.49 μM [135]. Other interesting examples of enantioselectivity were observed for other enantiomeric pairs of CDXs, such as CDX **64** [135,136]. In addition, in vitro DNA crosslinking ability was assessed for the active compounds and the results correlated with in vitro cytotoxicity [136]. It was inferred that a possible mechanism of action may involve induction of DNA crosslinking activity. Nevertheless, other mechanisms cannot be excluded and must be explored in further studies.

Other enantioselectivity studies associated with biological activity of CDXs were performed, specifically the in vitro and in silico inhibition of cyclooxygenases (COX-1 and COX-2) enzymes [140]. All tested CDXs inhibited both enzymes being, in general, the inhibitory effects similar. The only exception was the enantiomeric pair of CDX **70**, being the (*R*)-enantiomer more active at inhibiting COX-2 than COX-1 (Figure 16). The inhibitory effects for COX-1 demonstrated to be dependent on the stereochemistry of the compounds. Enantioselectivity was also observed for COX-2 inhibition being the CDXs (*S*)-**70** and (*S*)-**66** the more active compounds (Figure 16) [140].

Regarding that some CDXs have molecular moieties structurally very similar to some kind of local anaesthetics, the ability to block compound action potentials at the isolated rat sciatic nerve was also investigated [137]. The CDXs (*S*)-**70**, (*S*)-**67** and (*S*)-**68** (Figure 16) were chosen for biological evaluation and the results suggested that the nerve conduction blockade might result predominantly from an action on Na^+^ ionic currents. It was also investigated if the CDXs could prevent hypotonic haemolysis on rat erythrocytes. However, these data suggested that all tested CDXs caused no significant protection against hypotonic when applied in concentrations high enough to block the sciatic nerve conduction in the rat [137].

Additionally, considering that lipophilicity is one of the most important drug-like properties having a great impact in both pharmacokinetics and pharmacodynamics processes, the lipophilicity of a series of CDXs inferred by the partition between octanol and aqueous phase were assessed using four different methods: computational methods, vortex-assisted liquid-liquid microextraction coupled with LC, reversed-phase thin layer chromatography, reversed-phase LC, and biomembranes model by the partition between micelles and aqueous phase [141]. The methodologies and data gathered in this study allowed a better understanding of lipophilicity of this important class of compounds, which highlights the need to have different lipophilicity measurements to take conscious decisions in the hit to drug optimization process.

Different types of CSPs, namely Pirkle type, macrocyclic antibiotic and polysaccharide-based, were considered for quantification of the enantiomeric ratio, to explore the structure-enantioresolution relationship (SER) as well to investigate the chiral recognition mechanisms of the synthesized libraries of CDXs. All explored CSPs were suitable for quantification of the enantiomeric ratio of the majority of CDXs of all synthesized libraries. The resolution of seven enantiomeric pairs of CDXs on (*S*,*S*)-Whelk-O1 and L-phenylglycine CSPs was systematically investigated using multimodal elution conditions (normal, polar-organic, and reversed-phase) [142]. The (*S*,*S*)-Whelk-O1 CSP, under polar-organic conditions, demonstrated a high power of resolution for the CDXs possessing an aromatic moiety linked to the stereogenic center, the enantioselectivity (α) and resolution (Rs) ranged from 1.91 to 7.55 and from 6.71 to 24.16, respectively. The chiral recognition mechanisms were investigated for (*S*,*S*)-Whelk-O1 CSP by molecular docking and were in accordance with the experimental chromatographic parameters. The simultaneous π-π interactions that may be established by the phenyl group at the stereogenic center as well as the xanthone scaffold with the selector demonstrated to be essential for enantioselective recognition [142]. Lately, 32 new CDXs were investigated on (*S*,*S*)-Whelk-O1 CSP only in polar organic mode with acetonitrile/methanol (50:50 *v/v*) as mobile phase. The values of α and Rs ranged from 1.41 to 6.25 and from 1.29 to 17.20, respectively. Generally, the (*R*)-enantiomer was the first to elute. All the enantiomeric pairs were enantioseparated, even those without an aromatic moiety linked to the stereogenic center. The chiral recognition mechanisms by molecular docking were consistent with the chromatographic parameters and elution orders. Several structural requirements were revealed as being important for enantioselectivity: the xanthone tricyclic ring aligned with phenanthryl moiety of the (*S*,*S*)-Whelk-O1 selector in order to maximize π-π stacking interactions; the aromatic ring of the chiral moiety of xanthone derivatives established π-π stacking interactions with the 3,5-dinitrophenyl group of the selector and H-bonding interactions between the xanthone carbonyl and the NH amide of the selector [143].

In a study considering macrocyclic antibiotic CSPs for determination of the enantiomeric ratio, to explore the SER and the chiral recognition mechanisms by molecular docking, fourteen new CDXs were evaluated in Chirobiotic T, Chirobiotic TAG, Chirobiotic V and Chirobiotic R under multimodal elution conditions. The best α and Rs were achieved on Chirobiotic R and Chirobiotic T CSPs, under normal elution conditions, with Rs ranging from 1.25 to 2.50 and from 0.78 to 2.06, respectively. The chiral recognition mechanisms by molecular docking were able to estimate the resolution for CDXs with a success rate of 92%. The calculations also showed that each macrocyclic antibiotic CSP exhibits rather different binding patterns. The shape and the surface of the chirobiotic CSP, the general chemical structure and not only the position of the stereogenic centers of the CDXs played an important role in the overall retention of the enantiomers by the CSPs [144]. Recently, the same four macrocyclic antibiotic CSPs were used for a systematic study of enantioseparation of thirty-one new CDXs, confirming the applicability of these CSPs for the enantioseparation of this class of compounds [145]. It was found that their applicability increased considering the complementary profile in enantioselectivity of the different macrocyclic antibiotic CSPs. Higher resolutions were obtained for the CDXs evaluated compared to our previous work [143], and additional information about chiral recognition mechanisms was provided [145].

Regarding polysaccharide-based CSPs, the SER of twelve enantiomeric pairs of CDXs were investigated on *tris*-3,5-dimethylphenylcarbamates of cellulose and amylose and *tris*-3,5-dimethoxyphenylcarbamate of amylose under multimodal elution conditions. All the enantiomeric mixtures of CDXs were enantioseparated with very high values of α and Rs, ranging from 1.43 to 12.41 and from 1.48 to 10.29, respectively. Amylose *tris*-3,5- dimethylphenylcarbamate CSP under polar organic elution conditions presented the best results. The influence of the mobile phases and structural features of the CSPs and CDXs on chiral discrimination were also discussed. Considering the overall results, the chiral recognition of CDXs on polysaccharide-based CSPs depends not only on the nature of the CSPs but also on the mobile phase composition. The chemical bridges of the CDXs, the substitution of the xanthone scaffold, as well as the position of the stereogenic center had great influence on the enantioselectivity [143].

Recently, the SER, the chiral recognition mechanisms, and binding of CDXs on human serum albumin (HSA) CSP were also investigated (values of α and Rs ranged from 1.40 to 9.16 and 1.51 to 4.97, respectively). Percentages of binding ranged from 79.02 to 99.99%. The highest affinity values were achieved for the CDXs bearing bulky alkyl or aryl groups in the chiral side chain, suggesting that the hydrophobicity of the CDXs was important for HSA binding process. A good agreement (71%) between docking studies and experimental results was achieved, indicating that CDXs fit within the hydrophobic pocket of subdomain IIIA [146].

**Figure 18:**
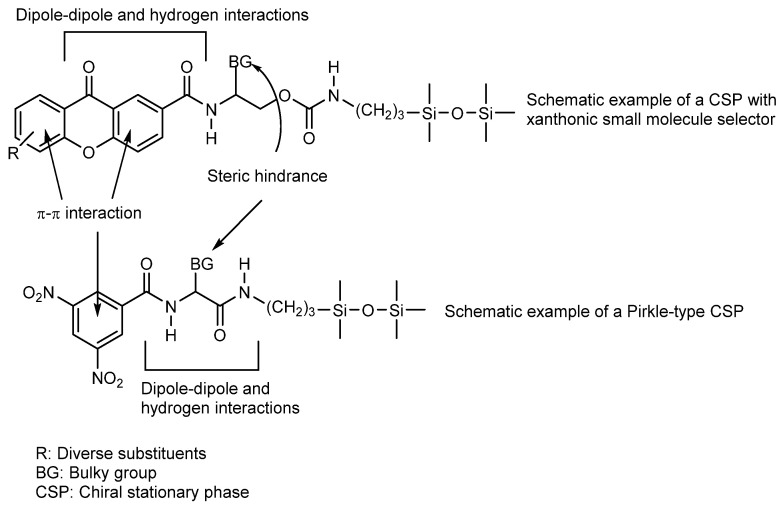
Schematic comparison of the general structure of a Pirkle’s CSP with a general xanthone small molecule as a selector.

In fact, the excellent results of SER of CDXs with a diversity of CSPs justify the investigation of CDXs as selectors for cLC. Thus, in addition to the interesting biological activities, CDXs have also been explored as selectors for CSPs [68,69,147]. CDXs possess all the necessary attributes to constitute good selectors for cLC due to the 3D quasi-planar structure and peculiar electronic properties of the xanthone scaffold and the diverse association with a variety of functional groups and chiral moieties, what results in structural characteristics that afford the establishment of different types of interactions as well as the three-dimensionality, factors that influence the enantioselectivity, similarly with the basic scaffold of Pirkle-type CSPs (Figure 18) [148]. Accordingly, six CDXs in enantiomerically pure form were covalently linked to silica gel through a spacer yielding the corresponding CSPs. The synthetic methodology involved only a few steps and the reactions provided good yields. These new CSPs are based on a completely different scaffold of small molecules of Pirkle’s CSPs [148]. The resolution capability of these CSPs was evaluated using different chemical classes of chiral compounds under multimodal elution conditions. A library of CDXs was also evaluated in order to explore the principle of reciprocity as well as the chiral self-recognition phenomenon. In general, the CSPs provided high specificity for the enantiomeric mixtures of CDXs evaluated, mainly under normal elution mode [69,147].

Recently, from the development of new CSPs for cLC based on the most promising CDXs as chiral selectors, twelve new CSPs were successfully prepared starting from suitable functionalized small molecules including xanthone and benzophenone derivatives. The chiral selectors comprise one, two, three, or four chiral moieties. The evaluation of the new CSPs was done with different classes of chiral compounds, including a library of CDXs. Specificity for enantioseparation of some CDXs was observed. Besides, assessment of chiral recognition mechanisms was performed by molecular docking approach, which were in accordance with the chromatographic parameters. X-Ray analysis was also used to establish the structure of a chiral selector 3D. Excellent values of α (5.08) and Rs (3.13) were obtained for 4-methoxy-α- methylbenzylamine, in reversed-phase mode, using CH_3_CN/H_2_O/TEA: 80/20/0.01 as mobile phase. Moreover, enantioselectivity was also obtained for other analytes, including the commercial amines *N*-benzyl-1-(1-naphthyl)ethylamine and alanine-2-naphthylamide, although with low resolution [68].

Our group provided a significant contribution synthesizing a bioinspired library of CDXs, through a very convenient synthetic strategy, and exploring the enantioselectivity in biological activities for this class of compounds. Some CDXs proved to act as blockers of sciatic nerve transmission, as inhibitors of growth of human tumor cell lines and cyclooxygenase enzymes; in some cases, being the activity dependent on the stereochemistry. Additionally, enantioseparation studies and enantiomeric purity evaluation of the CDXs have been conducted using different types of CSPs, namely Pirkle-type, macrocyclic glycopeptide-based, polysaccharide-based, and HSA CSPs. Besides the potential as new drugs, CDXs present structural features with interest as chiral selectors for CSPs in LC. Analytical application for these small molecules as chiral selectors for LC was discovered by your group for the first time.

### 2.7. Thioxanthones

**Figure 19:**
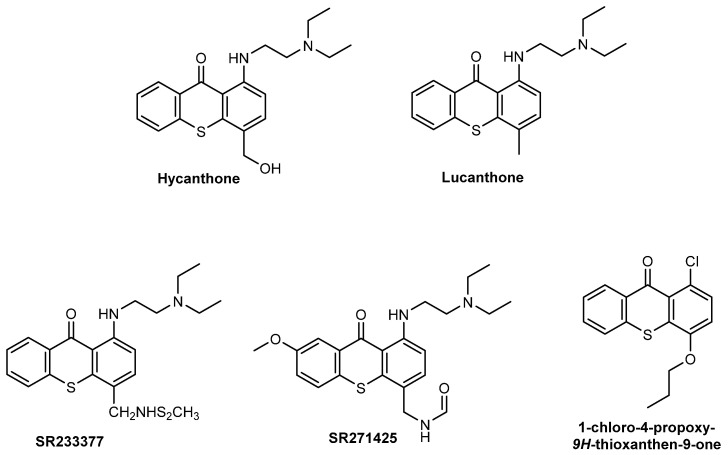
Structures of historically relevant thioxanthones.

Thioxanthones are isosters of xanthones. Structurally, they are *S*-heterocycles with a dibenzo-γ-thiopyrone scaffold, being described as an important class of molecules for their diverse biological properties, particularly their antitumor activity [126,149,150]. Their tricyclic scaffolds and the biological activities profile have led to the definition of thioxanthones as privileged structures, similarly to what happens with xanthones [98]. The first thioxanthones described as potential antitumor agents were hycanthone [151] and lucanthone [152], used as antischistosomal drugs (Figure 19). They were also described as antitumor agents with several mechanisms of action: DNA intercalation, inhibition of nucleic acid biosyntheses, inhibition of topoisomerases and apurinic endonuclease 1 [153]. Nonetheless, they were promptly withdraw due to mutagenicity [154] caused by the methylene linked to C-4 [155]. Thereafter, other thioxantones such as SR233377 [150,156,157] and SSR271425 [157,158] were tested in clinical trials as antitumor agents but were found to be cardiotoxic [159] (Figure 19). Despite that, the photoinitiator 1-chloro-4-propoxy-9*H*-thioxanthen-9-one was described as being safe and not genotoxic (Figure 19), according to GHS and to Directive 67/548/EEC. Consequently, our group considered that the design of thioxanthones without a methyl group or a methylene bridge at C-4 (lucanthone, hycanthone, SR233377, and SR271425), but with a propoxyl group at C-4, such as 1-chloro-4-propoxy-9*H*-thioxanthen-9-one, would be a suitable starting point for the discovery of new potential anticancer drugs.

However, we were aware that anticancer drugs often have low efficiency in the treatment and cure of cancer due to the acquired or intrinsic resistance of tumor cells to these agents [160]. Indeed, resistance to one drug often implies resistance to a series of different drugs, which are structural and functionally very distinct, the designated multiple drugs resistance (MDR) [160]. The MDR is heterogeneous, involving a combination of mechanisms, such as alteration of membrane transports [160]. P-glycoprotein (P-gp) or ABCB1 is the best characterized efflux pump implicated in MDR [161].

P-gp is an apical membrane transporter whose aim is to protect cells against xenobiotics and cellular toxicants. The overexpression of P-gp confers significant resistance to a wide variety of neutral and cationic hydrophobic chemotherapeutic substrates [162]. P-gp inhibitors generally have a logP of 2.92 or higher, a long chain to strengthen the link to P-gp; and at least one protonable nitrogen that can establish ionic and ion-dipole bonds [163,164,165]. Tariquidar and zosuquidar, belonging to the 3rd generation P-gp inhibitors, are examples of compounds that respect those characteristics [166]. Therefore, we concluded that antitumor agents that are P-gp inhibitors by themselves could represent a good first step toward the development of multi-functional drugs called dual-activity agents. Simultaneous modulation of multiple biological targets can be beneficial for the treatment of diseases with complex ethiologies such as cancer. Recently, there has been a paradigm shift in drug design towards the development of multifunctional drugs, which means that a compound can reach multiple targets [167,168].

**Figure 20:**
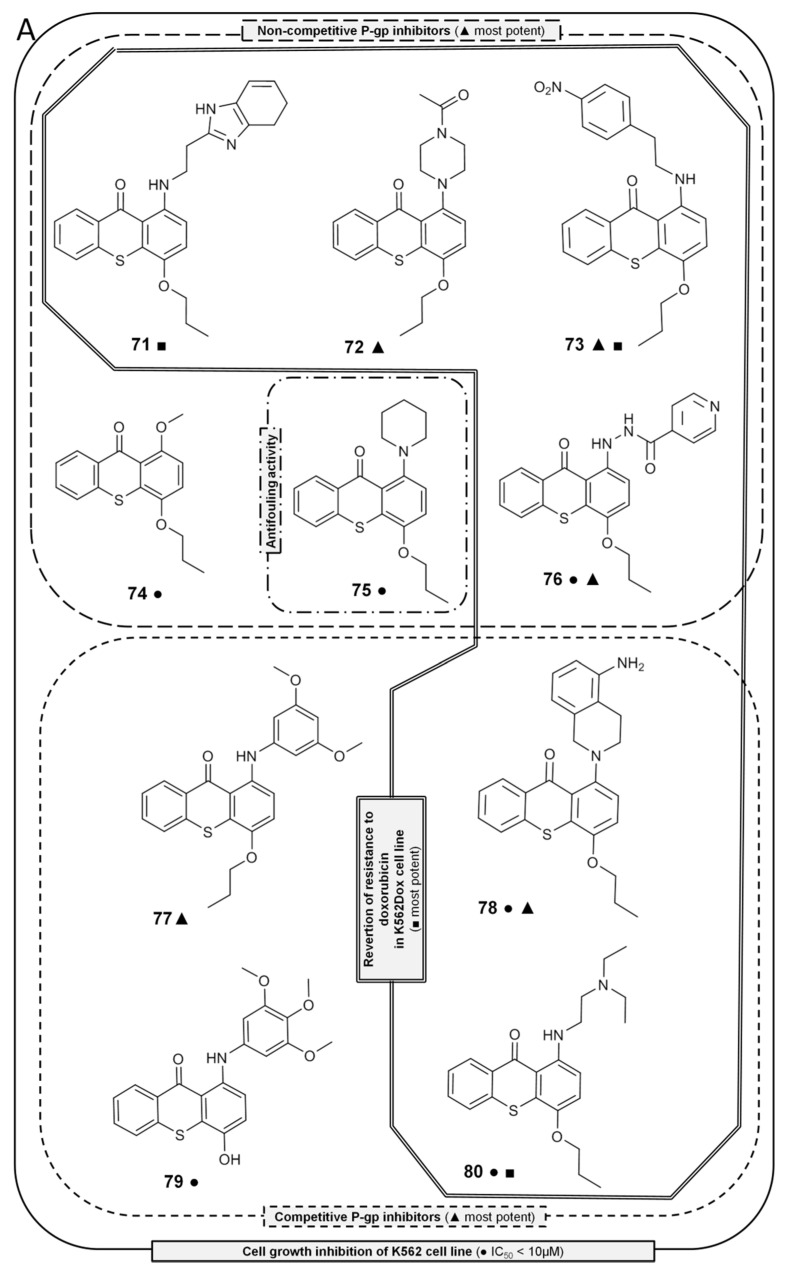

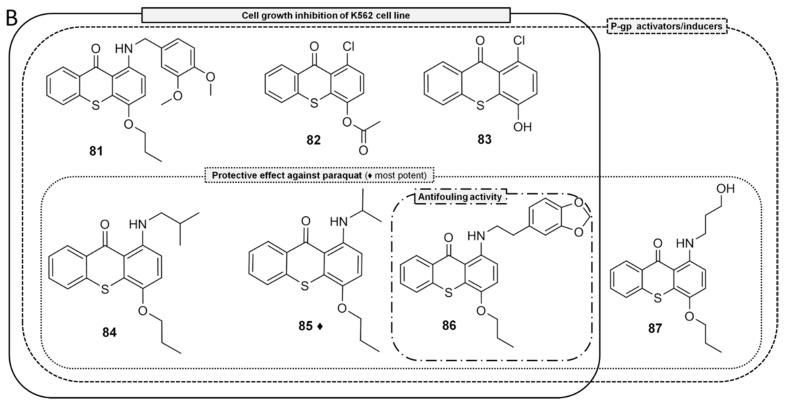
Thioxanthones and their biological activities: (**A**) Competitive and noncompetitive P-gp inhibitors. (**B**) P-gp activators/inducers.

Thus, our aim was to design new dual ligands that would have anticancer activity and simultaneously avoid MRD trough P-gp inhibition, by hybridizing a thioxanthone scaffold (antitumor activity) and an amine (group present in P-gp inhibitors). In fact, next generation of drugs will focus on more than one target, because this will really accelerate progress against complex diseases [169]. A docking study was performed using a dataset of approximately 1000 virtual C1 aminated thioxanthones and two human P-gp models. Lipinski rule of five and other features described as important for P-gp inhibition, such as logP value of at least 2.92 or higher [164,165] were applied as queries to the initial library of aminated thioxanthones. The molecules that passed through these filters were docked into the transmembrane domains (TMD) and nucleotide binding domains (NBD) of P-gp. Twenty-seven thioxanthones with the highest affinity towards P-gp were then synthesized by C(1)-N Ullmann cross coupling using conventional heating at 100 °C for 48h or MW-promoted heating at 205 °C for 50 min, in presence of K_2_CO_3_ base. Dealkylation was performed using boron bromide, BBr_3_ [170]. Secondary thioxanthonic products of the performed reactions without an amine at C-1 were also included in the in vitro studies. After purification and structural elucidation, a rhodamine-123 (P-gp substrate) accumulation assay [171] was performed in order to detect which thioxanthones were modulating P-gp activity. In this assay, two chronic myeloid leukemia cell lines were used, a sensitive cell line (K562) and a resistant cell line that overexpresses P-gp (K562Dox). The most potent P-gp inhibitors were **71**, **72**, and **73**, which increased the accumulation of Rh123 similarly to the known P-gp inhibitor quinidine (Figure 20). In order to determine how those compounds were interacting with P-gp, the measurement of their effect on the rate of P-gp ATP hydrolysis was performed to differentiate between thioxanthones that were increasing the P-gp-mediated ATP hydrolysis (competitive inhibitors or substrates) and thioxanthones that were diminishing P-gp-mediated ATP hydrolysis (non-competitive inhibitors) [170,172,173]. From the compounds that had caused accumulation of rh123 in the cells, 25% were acting as competitive and 75% as non-competitive inhibitors of P-gp (some examples are represented on Figure 20A). Thioxanthones **72**, **73**, and **76** were the best non-competitive inhibitors of P-gp, blocking the ATPase activity, whereas **77** and **78** were the best competitive inhibitors of P-gp, stimulating the ATP hydrolysis and being themselves transported by the pump [170] (Figure 20). The results obtained for thioxanthonic derivatives [170] and several commercially available drugs [171,174] in the in vitro ATPase assay led to the establishment of a pharmacophore for competitive and non-competitive P-gp inhibitory activity. In order to access the antitumor activity against tumor cell lines, a cell growth inhibitory effect (sulphorodamine-B assay) of 27 thioxanthones was performed on K562 (chronic myeloid leukemia cell line) [170], MCF-7 (breast cancer cell line), NCI-H460 (non-small cell lung cancer, NSCLC), and A375-C5 (melanoma cell line) [175]. Concerning the K562 cell line, six compounds presented GI_50_ inferior to 10 µM (**74, 75**, **76, 78, 79**, and **80**) (Figure 20); and eight presented GI_50_ between 10 and 20 µM, with no significant decrease in cellular viability of MRC-5 (non-tumor cell line). The most potent cell growth inhibitor thioxanthone was 1-[[2-(diethylamino)ethyl]amino]-4-propoxy-9*H*-thioxanthen-9-one (**80**), with a GI_50_ of 1.9 µM. This compound has a 2-(diethylamino)ethylamine side chain at position 1, equally to lucanthone (Figure 19), the first thioxanthone described as a potential antitumor agent which reached phase II clinical trials for glioblastoma multiforme [153,176]. Concerning the solid tumor cell lines, two compounds, **80** and the thioxanthone C4 acetyl derivative **82** (Figure 20B) were found to be the most potent on the studied cell lines, presenting GI_50_ concentration lower than 10 µM [175].

As the most potent compound on the four tested tumor cell lines was **80**, this compound, along with its hydrochloride salt, **80·**HCl, were further investigated to discover its cellular mechanism of action. An MTT assay using MCF-7 cells showed that 6 h treatment with **80·**HCl was sufficient to interfere with the cellular metabolic activity, cell survival and proliferation; and for longer periods no recovery was observed, suggesting that the effect is irreversible. Besides, **80·**HCl was acting preferably as a cytotoxic rather than as a cytostatic compound. At 10 μM and higher **80·**HCl concentrations there were alteration in cellular morphology which were suggestive of apoptosis.

A TUNEL assay and a flow cytometry analysis following Annexin V-FITC and propidium iodide (PI) showed that treatment with 10 μM **80·**HCl induced apoptosis in approximately 50% of the cells analysed [175]. Besides, **80** decreases the viability of melanoma cells by modulation of autophagy, causing accumulation of autophagosomes and the formation of autophagolysosomes at the GI_50_ concentration. An increase in LC3-II levels was also observed, which was reverted by 3-methyladenine (3-MA) (an early stage autophagy-inhibitor) but further increased by E-64d/pepstatin (late-stage autophagy inhibitors). Lastly, 3-MA also reverted the effect of **80** in cellular viability; and may, therefore, serve as a lead compound for the development of autophagy modulators with antitumor activity [177].

As **80** was considered a hit compound, with in vitro antitumor potential by modulating autophagy and apoptosis in human tumor cell lines, the mechanism of action of **80**.HCl was further investigated using in vitro and mouse xenograft tumor models of non-small-cell lung carcinoma (NSCLC). It was discovered that **80.**HCl caused abnormal cellular cholesterol localization and reduced the growth of human NSCLC cells in vitro and of NSCLC xenografts in mice, without presenting toxicity to the animals [178]. As perturbations in cholesterol localization or trafficking have been shown to affect cellular viability, this results provide new insights into a promising new mechanism of action of **80**, which may be relevant for the development of anticancer therapeutic strategies which target cholesterol transport [178].

Being the antitumor and P-gp inhibitory mechanisms of action already explored individually, the most promising thioxanthones were further investigated on K562Dox cell line (derived from K562 by doxorubicin stimulated overexpression of P-gp) with the purpose of analysing if thioxanthones were effectively capable of reducing cell growth of a resistant cell line. Six of the compounds had a GI_50_ value inferior to 20 μM on K562Dox cell line, suggesting a potential dual mechanism of action: P-gp and cell growth inhibitory activity. Notably, **80** was 9-fold more potent than doxorubicin in this cell line. Furthermore, six compounds significantly decreased the doxorucibin GI_50_ value on K562Dox cell line. At 1 µM, **71** and **73** caused a high decrease in doxorubicin GI_50_ on K562Dox cell line but almost did not affect doxorubicin GI_50_ on K562 sensitive cell line (the effect is mainly due to P-gp inhibitory activity), whereas **80** caused a high decrease in doxorubicin GI_50_ on both cell lines (the effect is also due to **80** own cytotoxic activity) [170] (Figure 20).

As a large number of studies indicated that other ABC transporters besides P-gp are involved in the efflux of endogenous molecules and xenobiotics out of the cell, our group decided to investigate the interaction of thioxanthones with other transporters, such as the multidrug-resistance associated transporter 1 (MRP1 or ABCC1), MRP2 (or ABCC2, cMOAT), MRP3 (or ABCC3) and the breast cancer associated protein (BCRP or ABCG2, MXR) [179]. Undoubtedly, a characteristic of all generations of P-gp inhibitors is that they are frequently multi-target inhibitors of other ABC transporters, as described for tariquidar [180], as these pumps share structural and functional similarities. A colorimetric-based ATPase assay using membrane vesicles from mammalian cells overexpressing a selected human ABC transporter protein (P-gp, MRP1, MRP2, MRP3, or BCRP) was performed by our group. Thioxanthone **78** was able to inhibit all the tested pumps, and **71** was able to inhibit all the pumps except MRP2 [179].

One of the problems regarding P-gp inhibitors is the pharmacokinetic interactions due to the fact that they can also be substrates or inhibitors of cytochrome p450, that will require the use of a higher dose of the cytotoxic compound to achieve therapeutic success [179]. As CYP3A4 has wide range of substrates and it is the subfamily of CYP responsible for the phase I metabolism of 50% of drugs administered to humans, a CYP3A4 luciferin-based luminescence assay was performed. The tested thioxanthones interfered with CYP3A4 activity in vitro (inhibitors or substrates), which was in accordance with the in silico prediction. It was found that **71** and **78** presented IC_50_ values for CYP3A4 inhibition of approximately 1–2 μM, but the inhibition was almost total for concentrations close to 100 μM [179].

**Figure 21:**
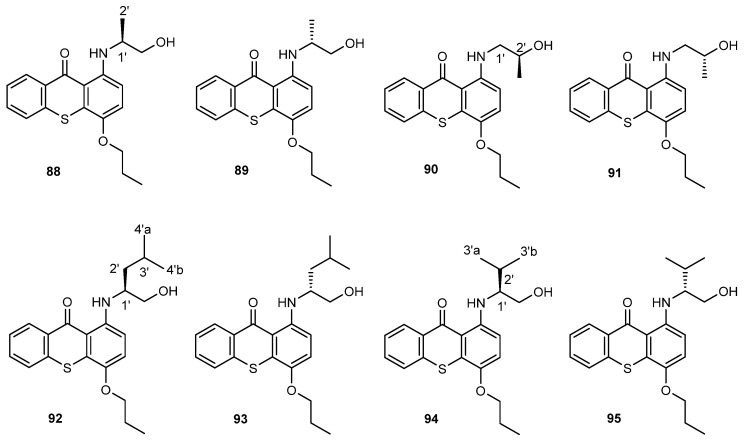
Chiral thioxanthones.

More recently, new chiral thioxanthones with aminated substituents at C-1 (2-amino-1-propanol, 1-amine-2-propanol, leucinol, and valinol) were synthesized in enantiomerically pure form in order to analyse the effect of the stereochemistry in P-gp modulation (Figure 21). All chiral thioxanthones at 20 µM significantly decreased the intracellular accumulation of a P-gp fluorescent substrate Rh123, suggesting an increment in the activity of the efflux pump. Therefore, these thioxanthones were shown to behave as P-gp activators, increasing the efflux of substrates without interfering with P-gp levels of expression. Moreover, (*R*)-1-((1-hydroxypropan-2-yl)amino)-4-propoxy-9*H*-thioxanthen-9-one (**89**), after 24 h of incubation, was also able to significantly increased P-gp expression (P-gp inducer) [181]. Concerning thioxanthones induction mechanism, one of the possibilities is the transcriptional activation of the MDR1 gene expression that involves the activation of the pregnane X receptor (PXR) [182,183]. Furthermore, no significant differences were detected in the activity of the enantiomeric pairs towards P-gp [181]. Still concerning P-gp induction in other organisms, using high throughput RNA sequencing, the transcriptional response of the filamentous fungus *Neurospora crassa* to thioxanthone derivatives with antitumor activity (**75**, **81**, and **86**) revealed that there is an induction of ABC transporters in that fungus, particularly atrb and cdr4, which is a common consequence of the treatment with (thio)xanthones [94]. Moreover, a group of genes were downregulated by the tested thioxanthones during oxidative stress [94]. While screening for potential P-gp inhibitors that increased the accumulation of known P-gp substrate Rh123, some of the tested thioxanthones caused the opposite effect, decreasing the Rh123 accumulation ratio. This effect is compatible with a possible P-gp activation, and such compounds are potential protectors against toxic effects induced by P-gp substrates [170]. Indeed, P-gp, besides being responsible for multidrug resistance of neoplastic cells to cancer therapy, is also constitutively expressed in many excretory and barrier tissues and, due to its wide substrate specificity, can play a crucial role in limiting the absorption and increasing the efflux of harmful xenobiotics. This defence mechanism can be very important at the intestinal barrier, as it can decrease the intestinal absorption of toxic xenobiotics [184].

Some thioxanthonic derivatives were tested for P-gp activation and/or induction, and its ability to protect Caco-2 cells from the herbicide paraquat induced toxicity was assessed. All tested thioxanthones (**83**–**87**) at 20 μM caused a significant increase in both P-gp expression (inducer) and activity (activator) as evaluated by flow cytometry using the UIC2 antibody and rhodamine 123, respectively (Figure 20). They also increased P-gp ATPase activity in MDR1-Sf9 membrane vesicles, indicating that all compounds were P-gp substrates. Paraquat cytotoxicity was significantly reduced in the presence of four thioxanthone derivatives (**83**–**86**), and this protective effect was reversed upon incubation with a specific P-gp inhibitor. In silico studies suggested a potential co-transport mechanism of thioxanthones and paraquat [184]. The most potent P-gp inducer and activator, **85**, can be considered a new and promising antidotal model against P-gp substrates cytotoxicity (Figure 20) [184]. Therefore, it was important to find a simple, rapid and economical method for the analysis and quantification of this thioxanthone. A reverse-phase high-performance liquid chromatography (HPLC) using a C18 column and a mobile phase composed of methanol-water (90/10, *v/v*) with 1% (*v/v*) TEA, at a flow rate of 1 mL/min, was used for chromatographic separation. The method was validated according to ICH and it was selective, as there were no interferences of endogenous compounds with the same retention time. Also, the developed method was linear for **85** concentrations between 0.5 and 150 μM. Therefore, this HPLC method could be used to detect trace amounts of these thioxanthone in biological samples [185].

Besides antitumor and ABC transporters inhibition, thioxanthones represent a class of compounds with many other potential and diverse biological activities. As several of these compounds were described as antimicrobial agents, our group decided to also study the potential of some of our in-house thioxanthones to fight biofouling. In this study, significant anti-settlement responses were identified in the presence of thioxanthone **75** (EC_50_ = 3.53 μM) and **86** (EC_50_ = 28.60 μM), concomitantly with low toxicity to mussel larvae (LC_50_ > 500 μM). Thus, adding to their already known pharmacological actions, these thioxanthones also revealed AF activity. Exposure of plantigrade larvae to compound **75** altered the abundance of proteins involved in major cellular metabolic processes as glycolysis/gluconeogenesis, lysosomal protein degradation, or proteins that are constituents of the cytoskeleton. With some of these constitutive proteins having an important role in metabolism and cellular functions, it is possible that **75** affects the metabolism of a broad number of organisms. However, in contrast to AF xanthones described in previous section (2.1, 2.2, and 2.5), thioxanthones **75** and **86** were found to be toxic (100% and 50% mortality at 50 μM, respectively) to *Artemia salina*. A QSAR model was constructed that will orient the design of future novel active compounds [55].

Thioxanthones have aroused enormous interest in the preceding decades because of their multidimensional health effects, as anticancer agents and MDR-reverter agents. Moreover, they are privileged structures and promising biomolecules due to their potential role as protective agents against toxic xenobiotics or due to its ability to inhibit settlement and growth of fouling organisms. These aspects and their impact on drug bioavailability have recently been reviewed in [186]. However, much more is still to be explored in the future concerning their applications in health and industry.

## 3. Conclusions

**Figure 22:**
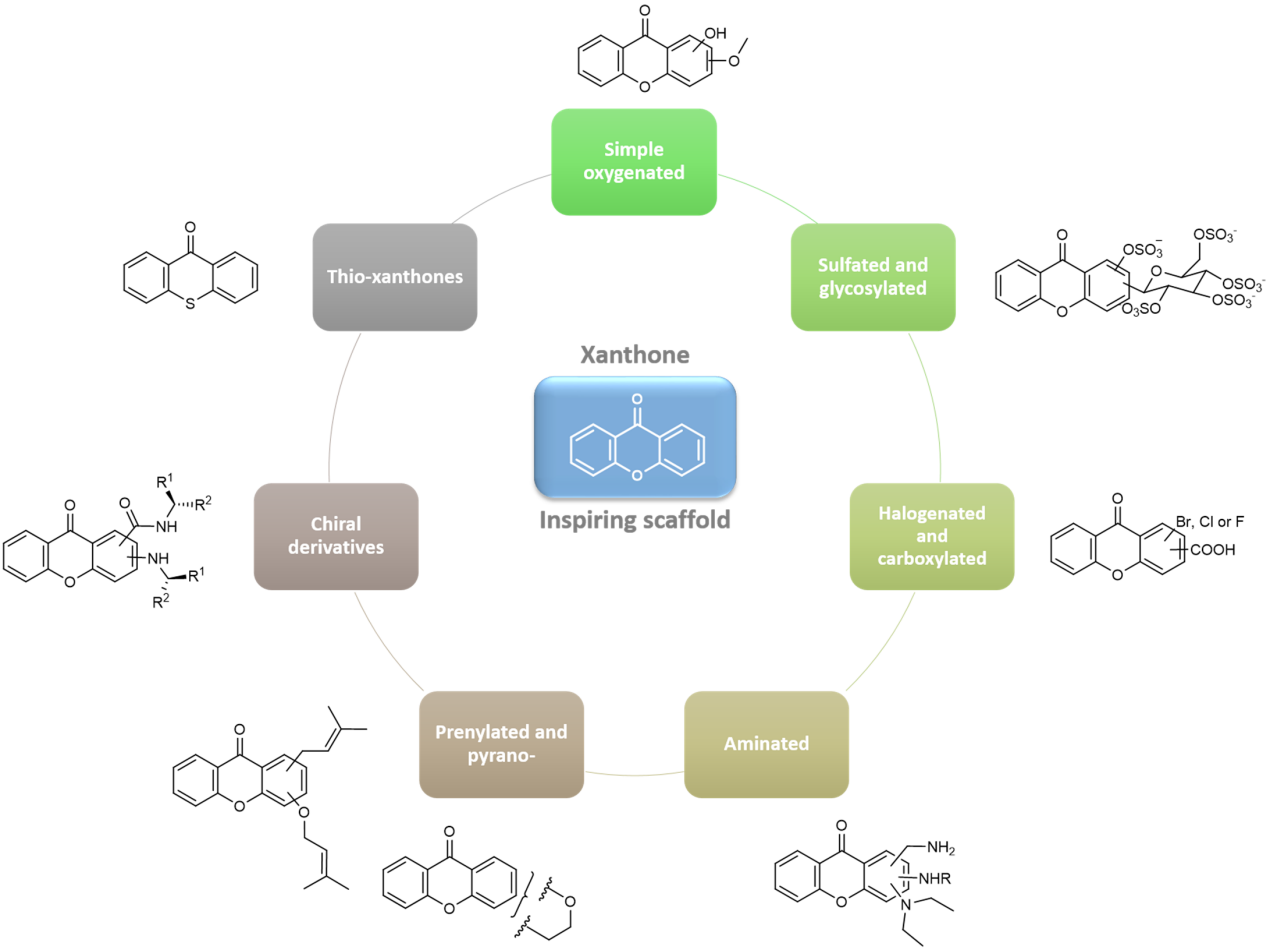
Our chemical “family tree” based on xanthone.

This review aims to give a perspective of the approaches and strategies used in our group to discover bioactive xanthone derivatives, and/or pharmacological probes, from natural and synthetic sources. Given the objective of this review, most of the references indicated throughout the text correspond mainly to the contribution of our group in this area.The hit compounds discovered for several targets and with diverse biological activities were highlighted and could be useful models to develop a vast number of heterocyclic privileged scaffolds. Not only is important to reach high-affinity hits but a strong investment should follow on safety, PK, process chemistry and comprehensive SAR understanding. Being focusing in the (thio)xanthonic scaffold is considered one of the strengths of our research group. However, we also aim for diversity and innovation by researching less explored areas (such as marine natural products), and most importantly by following a multi- and interdisciplinary approach, engaging chemistry of natural products, organic and medicinal chemistry, structure determination and purification, green chemistry, physicochemical properties and ADMET, validated in silico and HTS screenings, intellectual protection, drug delivery systems, etc. In summary, taking into account the chemical “family tree” based on xanthone (Figure 22), our group has:

(1) isolated new compounds from terrestrial and marine sources and/or analyzed species not yet studied with regard to their secondary metabolites;
(2) used Nature-based strategies to guide the synthetic ways whenever possible;
(3) synthesized these compounds for structure elucidation purposes to obtain them through “greener” methods and in quantities suitable for biological/pharmacological assays, as well as to obtain nature-based analogues with improved druglike properties;
(4) contributed with new data in the area of NMR and X-ray crystallography;
(5) evaluated several biological activities for different chemical families of xanthone derivatives; the main focus was in the area of antitumor and antimicrobial agents, especially taking into account MDR, which we believe should be pursuit in Academia; other areas of intervention include cardiovascular, antimalarial, anti-inflammatory, anti-obesity, hepatoprotection, etc. More recently, the area related to the discovery of environmental-benign AF agents was also explored;
(6) explored the mechanisms of action, SAR and structure-activity-properties-relationship (SAPR);
(7) formulated some compounds in nanoparticles, liposomes and proliposomes (drug delivery systems), especially with xanthones with potential antitumor activity;
(8) determined the drug-likeness of some hit compounds (ADMET assays), as this should be carried out as soon as possible in the pipeline of drug discovery and development;
(9) obtained several chiral derivatives of xanthones and studied enantioseparation and enantioselectivity with regard to various biological activities, as well as analytical applications as chiral selectors for liquid chromatography.

Finally, some of our hit xanthone derivatives may be emphasized as follows. As antitumor agents, the selective and poor water-soluble 1-carbaldehyde-3,4-dimethoxy-xanthone (**7**) and the drug-like P-gp dual inhibitor 1-[[2-(diethylamino)ethyl]amino]-4-propoxy-9*H*-thioxanthen-9-one (**80**) stand out; both proved their activity in patient derived-cells and in in vivo models, respectively. The first one still needs to be optimized due to unfavourable PK profile and the hit-to-lead optimization stage is ongoing based on the investigation of bioisosters of the aldehyde including ketones, ketoesters and boronic acids/esters. Since we have recently disclosed that the molecular mechanism of antitumor action of the dual inhibitor is related to the cholesterol transport, a balanced activity for both P-gp/antitumor activity can be tuned by structure-based design.

Concerning antimicrobial agents, marine-derived halogenated xanthones **28**–**31** were the most promising and efforts must be directed to improve solubility and to integrate chemical features that would overcome MDR such as the ones performed for other chemotherapies. This approach already allowed us to obtain chlorinated/aminated xanthone derivatives with broad activity spectrum also against parasitic protozoa. There are many examples of effective anti-infective drugs, which also do not obey the usual drug-likeness rules; therefore, considering the urgent need for new antimicrobial agents we can hope that marine-derived xanthones will be very useful models in this area of research. Sulfated groups have proven to be an important molecular feature for the recognition of several targets both in human (coagulation cascade) and in virus as well as to render mimetic derivatives of marine secondary metabolites with antifouling activity. Although the proof-of-concept for this class of xanthones is more advanced concerning safety, PK, and bioaccumulation profile, the chemical process to scale-up for kg applications is still a difficulty to surpass. Considering the analytical applications as chiral selectors for LC, the best enantioselectivity results were achieved for xanthone-phenylglycinol-derived CSPs. The next strategy to improve the design and obtain more efficient CSPs possessing wider applicability will be the preparation of selectors based on CDXs comprising one or more bulky aromatic moieties and polar groups to maximize π-π stacking and H-bonding, essential interactions for enantiorecognition.

## Data Availability

Not applicable.
